# A δ^2^H Isoscape of blackberry as an example application for determining the geographic origins of plant materials in New Zealand

**DOI:** 10.1371/journal.pone.0226152

**Published:** 2019-12-09

**Authors:** Kiri McComb, Shaerii Sarker, Jurian Hoogewerff, Alan Hayman, Russell Frew

**Affiliations:** 1 Department of Chemistry, University of Otago, Dunedin, New Zealand; 2 Global Proficiency Limited, Hamilton, New Zealand; 3 Faculty of Science & Technology, University of Canberra, Canberra, Australia; University of Maryland Center for Environmental Science, UNITED STATES

## Abstract

In this investigation, two previously reported precipitation δ^2^H isoscapes for New Zealand were used to develop a δ^2^H isoscape for blackberry (*Rubus sp*.) leaf. These isoscapes were calibrated using the measured δ^2^H values of 120 authentic blackberry leaf samples collected from across the country. A regression model based on environmental variables available for New Zealand was also determined to predict δ^2^H values measured from blackberry leaves without initially modelling the precipitation δ^2^H values. The three models were compared for their accuracy and precision when assigning 10 samples of blackberry leaves for their geographic location based on their measured δ^2^H values. One of the models based on a precipitation isoscape was similar in accuracy and precision of assignment to the model determined from the environmental variables and provides an approach for determining valid isoscapes for future plant materials.

## Introduction

Geographic source determination of biological materials based on water stable isotopes has been widely applied in the fields of ecology, anthropology and forensic science [1]. These isotope-based geographic assignment approaches are based upon querying a spatial model of the systematic changes of isotope ratios across a region, continent or the world. Such spatial isotopic models, or isoscapes as originally termed by West et al. [2], represent the changing relative abundances of stable isotopes (e.g. δ^2^H and δ^18^O values) due to changing environmental factors, such as differences in climatological, hydrological or ecological conditions.

The basic requirement for creating an isoscape is an isotopic dataset which represents the spatial area of interest. The Global Network for Isotopes in Precipitation (GNIP) was started in 1961 by the International Atomic Energy Agency in cooperation with the World Meteorological Organization (IAEA-WMO) [3,4]. GNIP provided spatial and temporal information regarding the distribution of ^2^H, ^18^O and ^3^H in precipitation from more than 1000 stations worldwide, among which 300 stations are engaged for stable isotopes [5]. This data set has allowed for the development of global isoscapes for the H and O isotopic composition of precipitation based on various empirical and geostatistical models [6–10].

While GNIP provides information to estimate global isotopic trends in precipitation, only two GNIP stations have been active in New Zealand [4,6]. These stations were active at Kaitaia (northern end of the North Island) and Invercargill (southern end of the South Island) from 1962–1994 and 1961–2015 respectively [4]. The information from these two stations was insufficient for the purpose of determining localised changes in the isotopic composition of precipitation within the country due to New Zealand’s variable climatic conditions [11]. The Cross-Departmental Research Project (CDRP) [12], funded by the New Zealand Ministry of Agriculture and Forestry (MAF), the New Zealand Department of Conservation (DoC) and the New Zealand Combined Law Agency Group (CLAG) was undertaken to measure a higher spatial and temporal resolution of δ^2^H values and δ^18^O values in precipitation across the country [12]. The CDRP project involved the collection of monthly precipitation from 58 sites in New Zealand and measurement of δ^2^H and δ^18^O values during the period August 2007 ‒ December 2009 inclusive [11–13]. The data obtained through the CDRP project have since been utilised to determine local precipitation isoscapes across New Zealand. Rogers et al. [14] reported an initial mean annual precipitation δ^2^H isoscape which was calculated using a multiple linear regression (MLR) approach based on elevation, mean annual temperature, and mean annual precipitation. The regression model reported by Rogers et al. [14] was shown to be significant with r^2^ = 0.86 and model parameter estimates were significant at p < α = 0.01. The determined precipitation isoscape was then linked to δ^2^H values measured from historic feathers from known locations by simple linear regression (r^2^ = 0.48) and utilised to investigate the potential geographic origins of feathers from historic Maori cloaks [14].

It has been shown that temporal variation in environmental parameters like rainfall, temperature and relative humidity, as well as geographic variation in temperature related to latitude and elevation, are drivers of the isotopic composition of precipitation [8]. The addition of measured environmental parameters as ancillary variables into isoscape models has shown to provide improved estimates of isotopic composition in precipitation [8] beyond using purely geostatistical approaches. Baisden et al. [11] reported a revised isoscape for δ^2^H values in precipitation across New Zealand based on the monthly precipitation δ^2^H values from the CDRP network, two geographic variables (latitude, elevation) and five daily climate variables (mean sea level pressure, minimum temperature, soil temperature at 10 cm, radiation and wind speed). The monthly weighted means of the daily climate variables were regressed against all available monthly δ^2^H values from the CDRP network. For monthly predictions, the model reported by Baisden et al. [11] yielded r^2^ = 0.43, for long term annual average predictions the model yielded r^2^ = 0.79.

The precipitation δ^2^H model reported by Baisden et al. [11] was used by Ehtesham et al. [13] to calibrate via linear regression to a model for determining the δ^2^H values of milk powders produced in New Zealand. The precipitation δ^2^H values predicted from the Baisden et al. [11] model were shown to be strongly related to the bulk δ^2^H value of whole milk powder (r^2^ = 0.86) as well as the compound-specific δ^2^H values of C14:0, C16:0 and C18:1 fatty acids extracted from milk powder. The precipitation model reported by Baisden et al. [11] allowed for making statistically valid predictions of isotopic composition in precipitation across specific time periods based on actual climate data [11].

Previous work using isoscapes for the geographic assignment of biological materials has shown the need for accurate precipitation isoscapes to underpin the semi-parametric Bayesian framework, which is widely used to determine the likely geographic origins [15]. Calibration of the precipitation isoscape is typically undertaken by determining functions which relate the precipitation isotope values to those of the biological material of interest [15–17]. These calibration functions are usually determined by the evaluation of empirical data collected on known origin samples of the material [15]. The δ^2^H values of cellulose derived from terrestrial plants have been shown to have a linear relationship with δ^2^H values in precipitation and can be very simply applied to calibrate a precipitation δ^2^H isoscape [1,18–25]. However, the relationship between the δ^2^H values of cellulose in plants and precipitation can be dependent on physical factors like evapotranspiration, temperature and humidity, and as such, some semi-mechanistic models have also been applied [24,25].

The utility of hydrogen isotope ratios as geographic origin indicators has been shown in numerous applications, including archaeology [26], food authentication [27] and forensics [24,28–31]. Booth et al. [29] utilised δ^2^H as one of several stable isotope indicators for the geographic origins of marijuana grown within and from outside of Alaska. While models based on precipitation δ^2^H values were determined by Hurley et al. [30,32] and utilised to indicate the origins of marijuana leaf in the conterminous United States. New Zealand specific examples include the previously mentioned application to bird feathers from historical Maori cloaks [14] and to milk and milk powders [13]; the δ^2^H values in beer and cider samples from New Zealand have been found to reflect the source water of precipitation and irrigation [33,34]. In studies by Holder et al. [35,36], the δ^2^H values of insect tissues were applied successfully to find information regarding their origin in New Zealand. Development of New Zealand specific isoscapes for plant materials could be utilised for forensic or biosecurity applications, including providing intelligence about growing regions in New Zealand or distinguishing imported plant materials. However, the limitation to this is the ability to obtain authentic samples of plant material with known geographical coordinates with which to calibrate a precipitation isoscape. Ideally, to calibrate the precipitation isoscape, species-specific isotope data from many plant samples covering the geographical extent of interest and inhabiting isotopically different locations would be obtained [16,37].

In this study, the δ^2^H values measured for blackberry (*Rubus sp*.) leaf samples from 130 different sites in New Zealand were collected. These samples were used in the development of a blackberry leaf isoscape as a function of δ^2^H values in precipitation. Blackberry is a mainly uncultivated species introduced to New Zealand that is prevalent across the whole country. It is deciduous, shedding its leaves every winter and is considered a moderately serious pest species in New Zealand as it is threatening native forests and wetlands [38]. This means blackberry is easily found and accessible for sampling across the country, allowing for a survey of plant material from a diverse number of environments.

The overall aim of this investigation was to determine an approach for the development of a plant material isoscape specific to New Zealand, which can be utilised for geographic assignment purposes. The precipitation isoscape models reported by Rogers et al. [14] and Baisden et al. [11] are used to provide the precipitation δ^2^H values for development of an isoscape which can be used to estimate blackberry leaf δ^2^H values. The resulting δ^2^H isoscapes for blackberry leaf are validated and compared to determine the more applicable base precipitation δ^2^H model. A blackberry leaf isoscape was also determined without prior modelling of the precipitation δ^2^H values by regression of the measured blackberry leaf δ^2^H values directly against environmental and geographic variables for further comparison. Prediction of the regions of likely origin for 10 blackberry leaf samples not previously utilised in the modelling is undertaken to provide an example of potential application. The approach taken could then be extended to other plant materials of interest, like marijuana for forensic purposes, or apples and kiwifruit as plant materials of economic and biosecurity importance to New Zealand.

## Methods

### Sample collection and preparation

**Fig 1:**
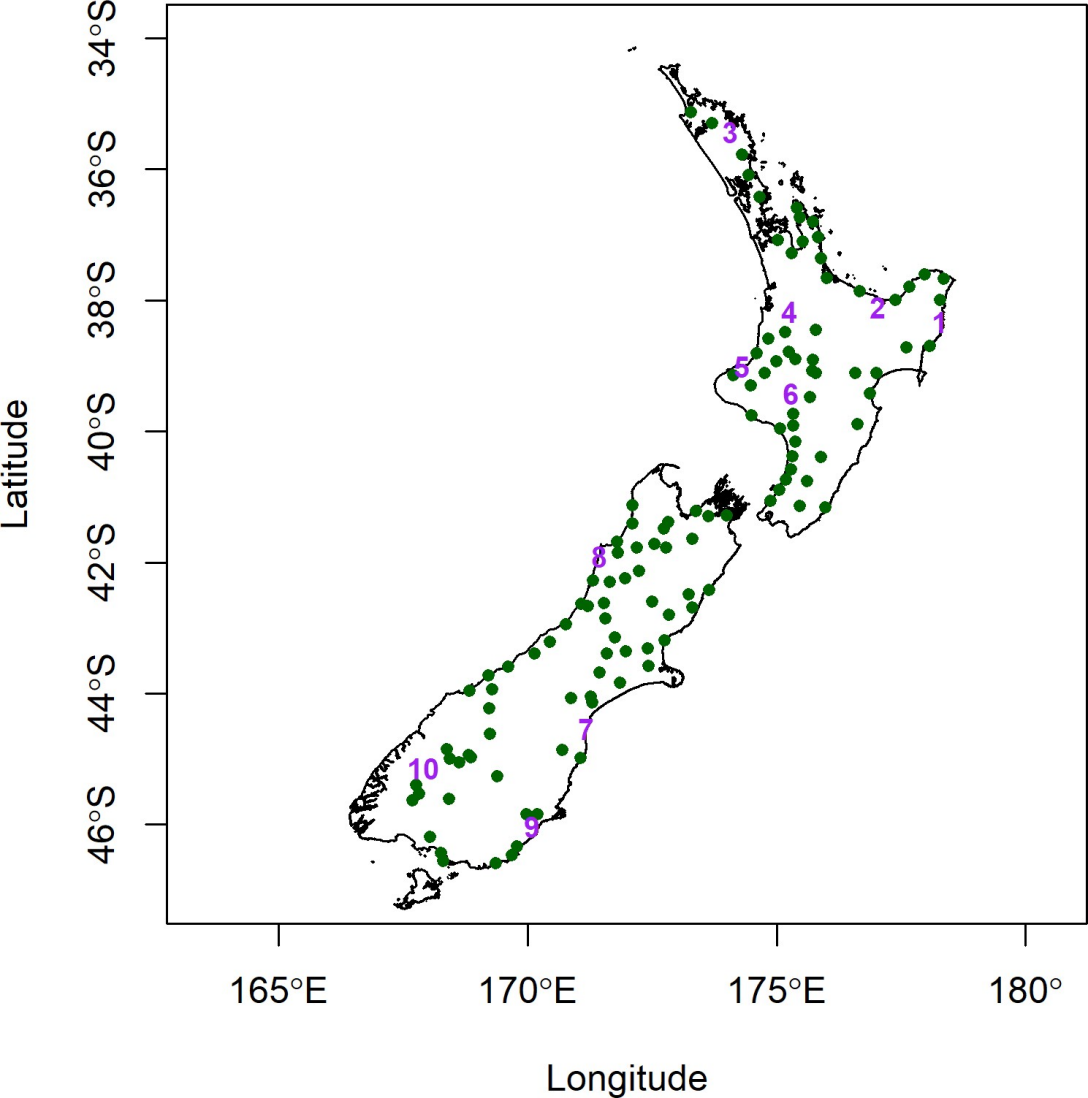
Map of the 130 sampled blackberry sites across New Zealand. The samples labelled 1–10 were further segregated as an external testing set for assignment.

A spatially representative set of blackberry samples (n = 130) from the North and South islands of New Zealand were obtained for the study over the period from October 2012 to January 2013. Approximately 30 g of each blackberry leaf sample was picked by hand and collected in a ziplock bag. The blackberry leaf samples were collected from recent, green and uniformly large leaves growing on the top of mature plants. Three individual plants were sampled at each site, and their geographic coordinates were recorded in decimal degrees (WGS84) using a mobile GPS unit (Garmin GPSmap 60CSx). The blackberry collection sites (Fig 1) were situated in areas under the authority of New Zealand local government or the New Zealand DoC. No specific permissions were required for access to these sites or for sample collection as these sites are open to public access and blackberry is not a threatened or protected species in New Zealand.

The samples were transported chilled to the laboratory where possible and stored refrigerated to prevent decomposition until they were able to be dried. The blackberry leaf samples were oven-dried at 30°C for 24 hours [39], which reduced the samples to ~68% of their original mass. The blackberry leaf samples were then stored at -20°C in ziplock bags within black polythene bags until further preparation was required. Approximately 1 g of blackberry leaf was sub-sampled into a MM 400 ball mill (Retsch, Haan Germany) container. The containers were milled overnight resulting in a fine flour. The samples were sieved (250 μm) to obtain a homogenous powder and stored in 10 mL sample vials in a desiccator ready for analysis.

### δ^2^H measurement

The *δ*^2^H value of the bulk leaf material was determined using two-stage equilibration [40,41]. The ambient temperature method with vacuum drying was used instead of the steam equilibration method in the analysis of the non-exchangeable *δ*^2^H value of the bulk leaf samples. This is because it has been reported that equilibration at ambient temperature produced more consistent and realistic non-exchangeable *δ*^2^H values in samples compared to the steam equilibration method [41].

Two sets of approximately 0.6 mg samples in triplicate were weighed into silver capsules (OEA Laboratories, Cornwall UK). A set of triplicates was loaded into each of two separate equilibration chambers along with triplicates of three quality control materials. The silver capsules were closed yet not crimped to allow the exchange of water vapour and effective drying prior to measurement. The samples were equilibrated in the separate equilibration chambers for six days with ~2 mL of different isotopic reference waters (High *δ*^2^H value water = +60 ‰ _VSMOW_ and Low *δ*^2^H value water = -262 ‰ _VSMOW_) at ~20°C after evacuating the desiccators for 2 min with a vacuum pump to remove air and ensure high concentration of water vapour in the chamber atmosphere.

After equilibration (>6 days), the samples were transferred to autosampler carousels that were placed inside aluminium chambers to isolate from the atmosphere. The samples were dried at 60°C for four days with online evacuation through a freeze-dryer [41]. All the samples were analyzed using a Delta V IRMS (Thermofisher Scientific) with attached TC/EA and Costech zero-blank autosampler. Measured values were reported vs VSMOW. The samples were standardized to reference materials USGS53 (*δ*^2^H_vsmow_ = +40.2 ‰), USGS47(*δ*^2^H_vsmow_ = -150.2± 0.5 ‰) and IAEA-CH-7 (*δ*^2^H_vsmow_ = -100.3 ‰). The quality control was conducted by applying three reference materials, Kudu Horn (*δ*^2^H_vsmow_ = -34.9 ± 0.6 ‰), Caribou Hoof (*δ*^2^Hvsmow = -156.4 ± 1.8 ‰) and RH-B (chicken feather) (*δ*^2^H_vsmow_ = 50 ± 2.8 ‰). Note: the original values for these keratin QC standards have been corrected according to the equation presented by Soto et al. [42]. The values presented here are our measurements. A set of the reference materials were measured at the beginning and at the end of every sample run. Duplicate IAEA-CH-7 aliquots were run after every 12 sample aliquots. Note that the QC materials used are all keratinaceous and not plant material. These were the only natural materials available at the time that had internationally accepted values for their non-exchangeable *δ*^2^H. As these materials have a very different matrix to the plant samples the results cannot be used for data correction, they are used to monitor the equilibration experiments and to show that consistent results are being obtained in each experiment. The lack of internationally accepted standards of the appropriate matrix and isotope value is a major problem for stable isotope analysis and one that needs to be addressed by the international community. The measured δ^2^H values are for the 130 blackberry leaf samples are reported in S1 Table.

### Statistical modelling

The resultant data set was split into a training set (n = 120) to investigate the variation of the δ^2^H values as a result of different climatic and environmental conditions in New Zealand. The remaining samples (n = 10) were set aside to be used as an external test set for assignment. The 10 test samples were selected using a stratified random sampling approach. The blackberry samples were grouped by region within New Zealand (defined by New Zealand Regional Council boundaries available from https://datafinder.stats.govt.nz/layer/92204-regional-council-2018-generalised/) and randomly selected from the different groups whilst ensuring no regional group was sampled more than once, to a maximum of 10 selected samples. This ensured the external test set included a spread of different locations and environments across New Zealand to be tested. The external test set was also chosen to represent a scenario similar to that of investigations in food authentication or forensics, where only a small number of independent suspect samples may be available for testing and are in need of being distinguished from particularly identified areas.

Two precipitation δ^2^H isoscape models have been published for New Zealand. The first model was reported by Rogers et al. [14], the second model was reported by Baisden et al. [11]. The reported underlying datasets for these models (CDRP [12], WorldClim [43] and VCSN [44]) were obtained, and the models calculated in line with each reported method to accurately reproduce each of the precipitation isoscapes as raster grids.

The CDRP data has been previously reported by Frew et al [12] and is available in S2 Table.

The precipitation δ^2^H isoscape model reported by Rogers et al. [14] utilised data for elevation, mean annual temperature and mean annual precipitation obtained from the WorldClim database as predictor variables. The WorldClim database currently provides open access to 19 bioclimatic variables as an average for 1970–2000 at 10, 5, 2.5 or 0.5 arc minutes spatial resolution for global land areas [43,45]. Rogers et al. [14] utilised an older version of the WorldClim database (version not reported) which was an average of bioclimatic variables over the period 1950–2000 with a resolution of 2.5 arc minutes [46]. The mean annual precipitation data were transformed using the base 10 logarithm before modelling.

The precipitation δ^2^H isoscape model reported by Baisden et al. [11] was based on multiple linear regression of 2 geographic variables (latitude and elevation), and 5 daily precipitation weighted climate variables (mean sea level pressure, soil temperature at 10 cm, radiation, minimum temperature and wind speed). The climate variables were determined from modelled daily climate data obtained from the Virtual Climate Station Network (VCSN). The VCSN is a climate database, developed and maintained by the New Zealand National Institute of Water and Atmospheric Research (NIWA) [44], that uses interpolation techniques to create a daily climate record at every point on a ~5 × 5 km grid covering all of New Zealand [47,48]. Daily climate variables were weighted by daily precipitation amounts to generate monthly weighted climate means averaged to cover the years 2007–2013, this includes the years of CDRP collection (2007–2009) through to the years of blackberry collection (2012–2013).

Each of these two isoscape models was utilised to determine the δ^2^H values for precipitation at the blackberry leaf collection sites, utilising an approach similar to that reported by Ehtesham et al. [13]. The average δ^2^H values in precipitation for grid cells within a 5 km radius of each blackberry sampling site were regressed against the measured δ^2^H values in the blackberry leaves. Each of the resulting simple linear regression equations, **Model 1,** which used values from the precipitation δ^2^H isoscape model of Rogers et al. [14] and **Model 2**, which used values from the precipitation δ^2^H isoscape model of Baisden et al. [11], were used to calibrate the precipitation isoscapes for estimation of δ^2^H values in blackberry leaves.

Calibration of precipitation isocapes to a biological material of interest is mainly undertaken due to a lack of the biological material δ^2^H values to model directly [1]. Given a reasonable number of biological material samples for this investigation (n = 120) an attempt at modelling the δ^2^H values of the blackberry leaves against the geographical variables of latitude, longitude (decimal degrees) and altitude (metres above sea level), as well as daily precipitation-weighted environmental variables from the VCSN database by multiple linear regression was also undertaken for comparison purposes (**Model 3**). Backwards stepwise selection of variables was undertaken following the method outlined in Baisden et al. [11] and the model resulting in the lowest Akaike Information Criterion (AIC) value was selected for use, taking into account the significance of variables entered into the model.

Posterior probability surfaces for geographical assignment were determined from each of the three different models using a semi-parametric Bayesian framework as has been reported in a number of studies and used in several platforms that have been developed for the determination of isoscapes, like IsoMAP and IsoriX [15,49]. In short, Bayes theorem is applied to evaluate the probability, *A*_*i*_ that a sample is from site *i* given that sample’s measured isotopic value, *δ*_*s*_, as shown in Eq 1.

$$P(A_{i}|\delta_{s})=\frac{P(A_{i})P(\delta_{s}|A_{i})}{\sum P(A_{i})P(\delta_{s}|A_{i})}$$ (1)

Where *P(A*_*i*_*)* is the prior probability associated with location *i* and, the summation is across all locations [15]. For this study, *P(A*_*i*_*)* was assumed uniform across all locations. *P(δ*_*s*_*|A*_*i*_*)* is evaluated as shown in Eq 2.

$$P(\delta_{s}|A_{i})=\frac{1}{\sqrt{2\pi \sigma_{i}^{2}}}e^{\left(\frac{-{\left(\delta_{s}-{\hat{\delta }}_{i}\right)}^{2}}{2\sigma_{i}^{2}}\right)}$$ (2)

Where ${\hat{\delta }}_{i}$ is the isoscape predicted value for the blackberry δ^2^H at location *i* and σ_*i*_^*2*^ is the total variance on the predicted sample value for site *i*. A similar approach to that outlined by Courtiol et al [49] was followed to determine σ_*i*_^*2*^, where this total variance depends upon (i) the prediction variance associated with the precipitation model, which varies spatially, (ii) the residual variance of the calibration, which is assumed constant, (iii) the prediction variance of the calibration model (which decreases with the size of the calibration dataset) given the predicted isoscape value at the candidate location and (iv) a covariance term between the prediction error of the calibration model given the predicted isotope value at the candidate location and the prediction error of the precipitation isoscape at this location.

Initial testing of the geographic assignment was undertaken using a repeated k-folds cross-validation approach. For each of 10 repeats, the 120 blackberry samples of the training set were split into 10 folds using random sampling without replacement. Each of the k folds in the repeat was used as a testing set while the remaining k-1 folds were used to redetermine the linear regression function. This allowed for 10 times 10 different folds to be tested (1200 individual tests) whilst ensuring every sample was tested an equal amount of times. A k = 10 fold approach was utilised as this has been empirically shown to limit bias of the test estimate introduced by removal of data from the model building process while incorporating a modest amount of variance [50]. The exact same determined folds were utilised to cross-validate and determine posterior probability surfaces from each of the three models in the investigation. The models were then used to determine the geographical assignments of the external test set of ten samples that had not been utilised in the model building step.

The determined posterior probability surfaces from the cross-validation and external test set were normalised so that all sample probabilities summed to 1 across the spatial domain. The surfaces were then rescaled between 0 and 1 for clarity and ease of comparison, similar to Wunder et al. [37] and Vander Zanden et al. [51]. The resulting surface consists of a range of probabilities where a value close to 0 is a low probability for geographic assignment of the test sample, and a value close to 1 is a high probability for geographic assignment. Areas with rescaled probability >0.667 were considered the likely origin of the test sample, based on the commonly used 2:1 odds ratio threshold reported in previous investigations [17,52].

The efficacy of geographic assignment for the different blackberry leaf δ^2^H isoscapes were compared using accuracy, precision and similarity metrics as described in Vander Zanden et al. [51]. Individual-level accuracy was evaluated as the relative change in probability between assignments at the known origin site and reflects whether the relative likelihood of origin increases or decreases at the true location when comparing one isoscape to another. Individual-level precision was evaluated as the change in the area of the posterior probability surface at a relative probability density equal to that at the known origin. This metric demonstrates whether the precision increases or decreases for an individual test sample when comparing one isoscape to another. Two-sided paired t-tests were used by Vander Zanden et al. [51] to determine if the mean individual level accuracy and precision values differ from zero. The similarity index reported by Vander Zanden et al. [51], calculates the number of shared cells between two assignment rasters to the total number of possible cells. A function of relative probability against similarity is determined for each individual which is then integrated to find the area under the curve as a value between 0 (no parts match) and 1 (all parts match exactly). This similarity index can be informally considered as the proportion of overlap of the two assignments over the entire range of relative probabilities [51].

All statistical modelling and assessments were undertaken using the R project for statistical computing [53].The R scripts used to conduct assignments and calculate accuracy, precision and similarity have been previously reported in Vander Zanden et al. [51]. A list of the R packages used in this investigation is provided in S3 Table.

## Results and discussion

### Model 1

The resulting parameters for Model 1, the simple linear regression of measured blackberry δ^2^H values against precipitation δ^2^H values using the model reported by Rogers et al. [14] are shown in Table 1.

**Table 1:** Linear regression parameters for Model 1.

	Estimate	Std. Error	t-value	Pr(>t)
Intercept	-53.84	2.92	-18.44	<0.0001
δ^2^H_precipitation_	0.75	0.07	10.63	<0.0001
RSE	8.6, 118 d.f.			
r^2^	0.49			

**Fig 2:**
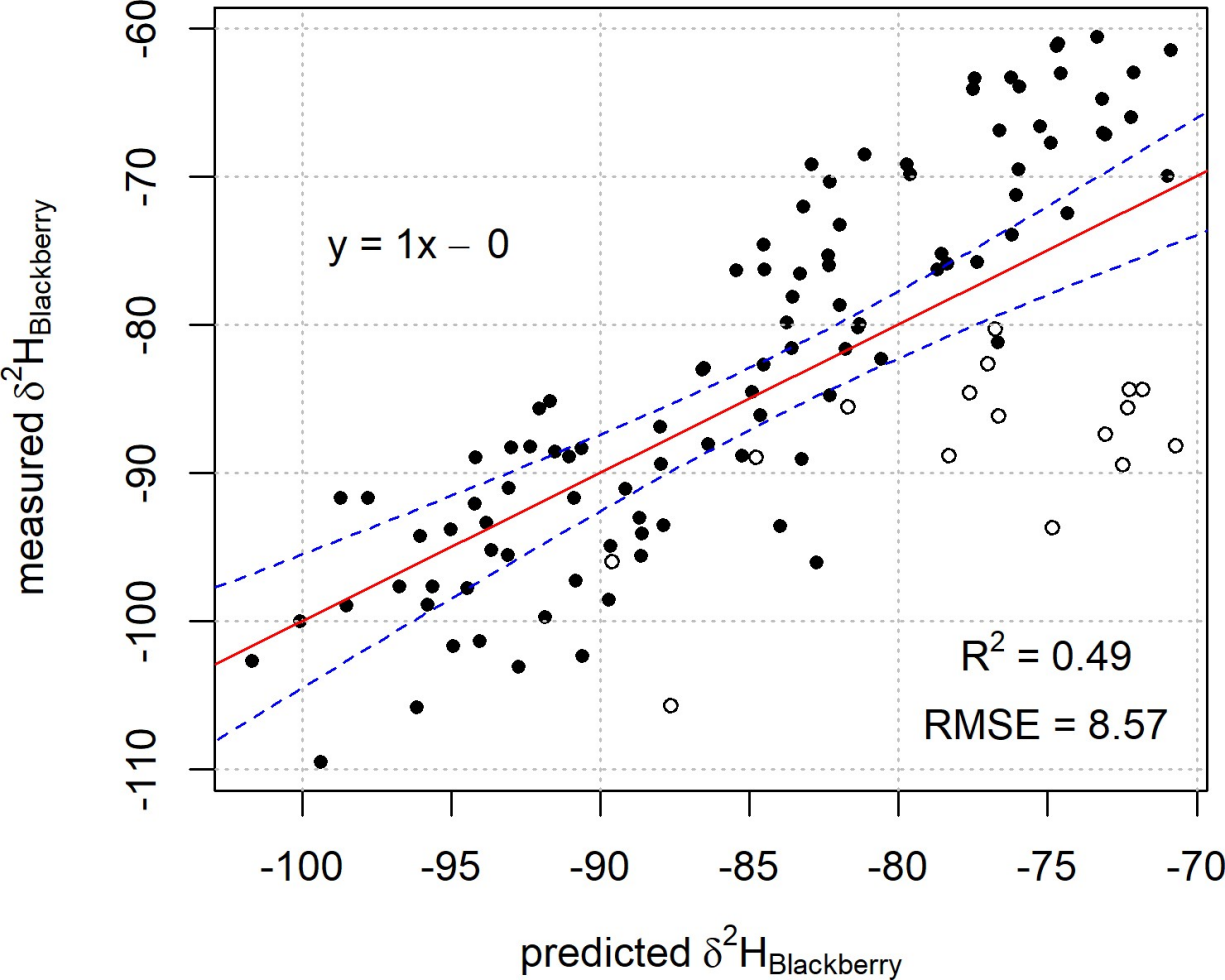
Plot of predicted blackberry leaf δ^2^H values based on Model 1 against the measured blackberry leaf δ^2^H values. The linear regression line of best fit is represented by the red line with 99% confidence intervals for the fit in blue. Samples from the West Coast region of the South Island are represented by open circles (○).

A plot of the regression result, measured against predicted δ^2^H values of blackberry leaf exhibits divergent non-linear behaviour at higher predicted values (Fig 2).

**Fig 3:**
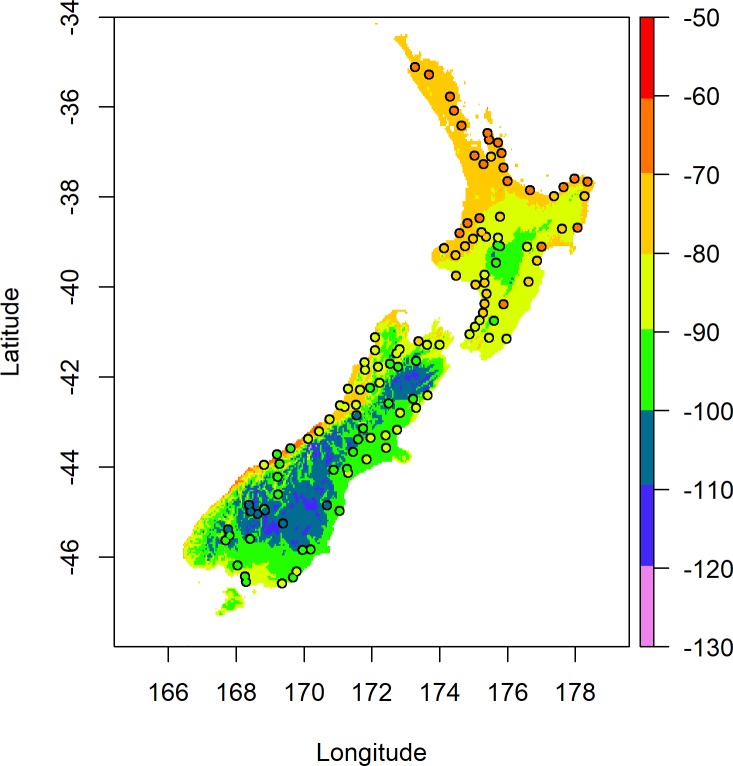
Predicted blackberry leaf δ^2^H values (‰) across New Zealand based on Model 1. Coloured points represent the measured δ^2^H values for blackberry leaves sampled from those locations.

This behaviour results in a root mean squared error of 8.6 ‰ and r^2^ of 0.49. Upon further investigation, the non-linear behaviour observed is a result of an approximately 10 ‒ 20 ‰ difference between the measured δ^2^H values of blackberry from the West Coast of the South Island (as shown by the open circles in Fig 2) and the predicted values for the same region derived from Model 1. The isoscape of model-derived predictions for blackberry leaf δ^2^H values based on Model 1 are shown in Fig 3.

It can be observed in Fig 3 that along the West Coast of the South Island and across the northern half of the North Island the predicted values do not agree well with the measured values, this is due to the difference observed in the underlying data for the West Coast of the South Island which exerts undue leverage on the regression and skews the prediction surface. The cause of the divergent behaviour observed in Fig 2 is surmised to be due to the temporal extent of the WorldClim data that was averaged into the precipitation δ^2^H model reported by Rogers et al. [14] which covers records from 1950–2000. Historically, a high amount of average annual precipitation has been recorded for the West Coast of the South Island (4000–5000 mm) over this period compared to the rest of New Zealand (1000–2000 mm). The result of using the linear calibration with the longer-term averages of the precipitation δ^2^H model reported by Rogers et al. [14] gives higher predicted δ^2^H values for blackberry leaves along the West Coast of the South Island compared to the measured values and lower predicted δ^2^H values for blackberry leaves from the upper North Island. A map of the residuals for the calibration model is shown in S1 Fig, which highlights the underestimation and overestimation of δ^2^H values in different areas of New Zealand. Assessment using a test for Moran’s I was significant at p < 0.01 confirming the presence of the observed spatial autocorrelation in the residuals. For the purposes of geographic assignment, the prediction variances were determined by cross-validation of the Roger’s precipitation δ^2^H model and Model 1 respectively. The residual variance of Model 1 was determined as 73.9 (s.d. of 8.6 ‰) during the model calculation, and as the precipitation model was able to be reproduced, the covariance terms were also able to be determined. The resulting map of variance for each grid cell ranges from 74 to 85 (s.d. of 8.6 ‰ to 9.2 ‰).

The repeated k-folds cross-validation for Model 1 resulted in, at the selected threshold level, 63% of true locations within the determined region of origin. This came with a mean precision of 74 351 km^2^.

The likely area of origin for each of the test set of 10 blackberry samples was determined using the assignment framework previously described. The figures showing the areas of likely origin are shown in S1 File. Of the 10 test samples, two of these samples (1, and 4) have a low determined probability at their actual location of origin. These test samples are located in the upper half of the North Island of New Zealand, one of the areas affected by the skewed linear calibration. The measured δ^2^H values for the test samples and those predicted by Model 1 are shown in Table 2 along with the difference between the two δ^2^H values (measured–predicted) as well as the scaled probability from the geographic assignment.

**Table 2:** Measured δ^2^H values for each of the test set blackberry leaf samples and the predicted values calibrated from Model 1. The difference between the values (measured–predicted) and the probability at the test sample origin from the geographic assignment are also shown.

Test Sample	δ^2^H_measured_ (‰)	δ^2^H_predicted_ (‰) Model 1	δ^2^H_measured_− δ^2^H_predicted_ (‰) Model 1	Assignment Probability Model 1
1	-67.8	-76.3	8.5	0.63
2	-70.1	-75.6	5.5	0.82
3	-65.7	-72.8	7.1	0.72
4	-66.3	-77.7	11.5	0.42
5	-70.8	-75.9	5.1	0.84
6	-86.1	-88.9	2.8	0.95
7	-89.8	-92.9	3.1	0.93
8	-75.0	-74.2	-0.8	0.99
9	-95.2	-93.0	-2.2	0.97
10	-95.9	-89.6	-6.4	0.76

This result reinforces the importance of having an appropriate precipitation isoscape model, which is related in process to the sample material to be investigated. In this case, due to the higher δ^2^H values estimated for the precipitation on the West Coast of the South Island and the leverage exerted on the linear regression, the estimates of Model 1 are skewed and not appropriate for the material being investigated.

### Model 2

Given that blackberry is deciduous, and sheds leaves in winter, leaf material will, at most, integrate δ^2^H values for a finite time period prior to sampling (depending on the time of season sampled). This suggests that a precipitation δ^2^H model based on long term averages may not be appropriate, and an approach which can provide δ^2^H values across a shorter time period will be more accurate [17]. This has been hypothesised and investigated previously by Vander Zanden et al. [51]. In this previous investigation, the biological materials of interest were monarch butterflies and Eurasian reed warblers. The resulting comparisons showed little to no improvement using a targeted short term precipitation isoscape when compared to a long term isoscape; however there was uncertainty around the effect of water memory assumptions used to estimate the shortened timeframe for monarch butterflies and acknowledgment that short term isoscapes may result in greater assignment efficacy in regions where greater annual variation of the isotopic composition of precipitation may occur [51]. New Zealand is subject to varying exposure from sub-antarctic and subtropical air masses resulting in a climate which is highly variable, and the subject of a more temporally refined isoscape precipitation model put forward by Baisden et al. [11]. The resulting parameters for Model 2, the simple linear regression of measured blackberry leaf δ^2^H values against precipitation δ^2^H values using the model reported by Baisden et al. [11] are shown in Table 3.

**Table 3:** Linear regression parameters for Model 2.

	Estimate	Std. Error	t-value	Pr(>t)
Intercept	-30.87	1.76	-15.52	<0.0001
δ^2^H_Precipitation_	1.31	0.04	30.66	<0.0001
RSE	4.0, 118 d.f.			
r^2^	0.89			

**Fig 4:**
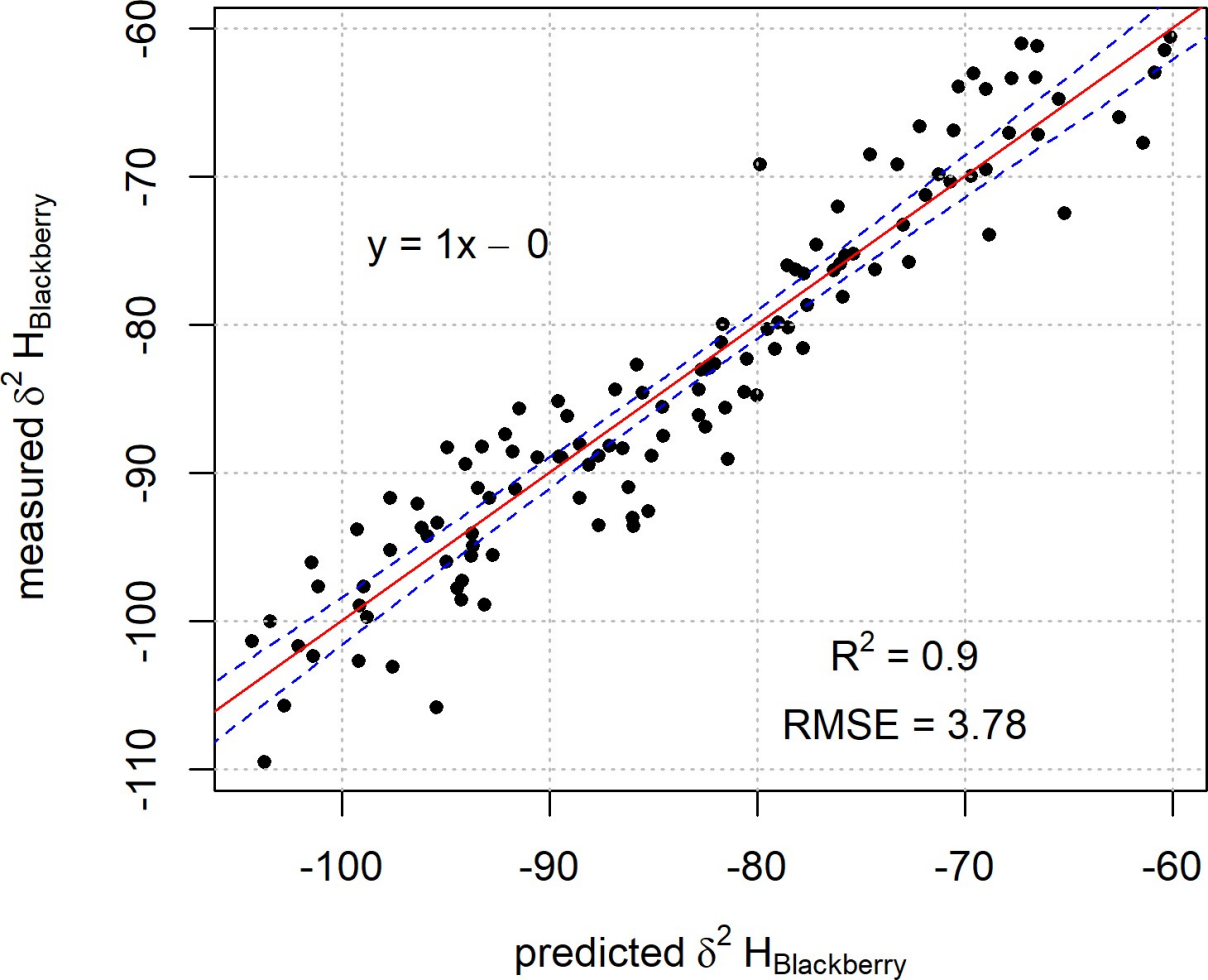
Plot of predicted blackberry leaf δ^2^H values based on Model 2 against the measured blackberry leaf δ^2^H values. The linear regression line of best fit is represented by the red line with 99% confidence intervals for the fit in blue.

The non-linear behaviour observed previously in Fig 2 is not present in the plot of the regression result for Model 2 (Fig 4).

This is possibly due to the precipitation model representing a more appropriate time frame for the incorporation of the precipitation δ^2^H into the sample material. This would suggest that a model based on a shorter timeframe is likely to be more representative of the integration of δ^2^H values into this type of material as it will not average in the large variances in climatic conditions experienced by New Zealand over longer periods.

**Fig 5:**
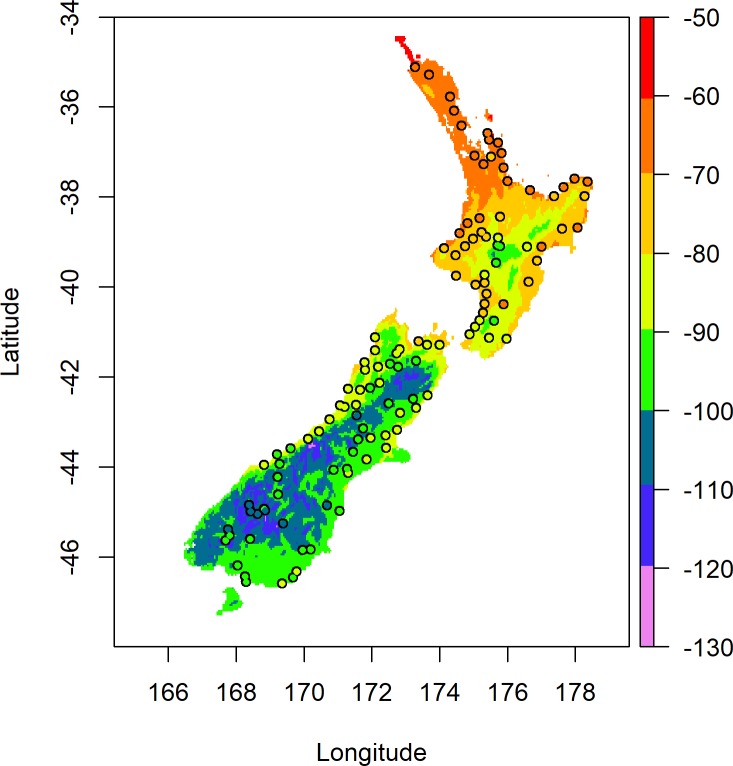
Predicted blackberry leaf δ^2^H values across New Zealand based on Model 2. Coloured points represent the measured δ^2^H values for blackberry leaves sampled from those locations.

The isoscape of model-derived predictions for blackberry leaf δ^2^H values based on Model 2 is shown in Fig 5.

It can be observed in Fig 5 that the predicted values empirically show better agreement with the measured values than was observed in Fig 3, especially along the West Coast of the South Island and across the northern half of the North Island. This observation is further supported by the smaller RMSE of 4.0 ‰ when the predicted results are compared to the measured results for Model 2 compared to the RMSE of 8.57 ‰ for Model 1. A map of the residuals for Model 2 is presented in S2 Fig, which shows a more random spread across the country than the residuals of Model 1. Assessment using a test for Moran’s I was not significant with p = 0.29, providing little evidence to reject the null hypothesis for the presence of spatial randomness in the residuals. For the geographic assignments, the prediction variances were determined by cross-validation of the Baisden precipitation model and calibration model, respectively. The residual variance of Model 2 was determined as 14.38 (s.d of 3.8 ‰) during the model calculation, and as the precipitation δ^2^H model was able to be reproduced, the covariance terms were also able to be determined. The resulting map of variance for each grid cell ranges from 14 to 24 (s.d. of 3.7 to 4.9 ‰).

The repeated k-folds cross-validation for Model 2 resulted in, at the selected threshold level, 68% of true locations within the determined region of origin. This came with a mean precision of 31 002 km^2^.

The likely area of origin for each of the test set of 10 blackberry samples was also determined for this model. The figures showing the areas of likely origin are shown in S2 File. 9 of the 10 test samples have a high determined probability at their actual location of origin with the true location of test sample 8, having a probability of 0.56. The measured δ^2^H values for the test samples and those predicted by the model based on the precipitation isoscape from Baisden et al. [11] are shown in Table 4 along with the difference between the two δ^2^H values (measured–predicted) as well as the scaled probability from the geographic assignment.

**Table 4:** Measured δ^2^H values for each of the test set blackberry leaf samples and the predicted values calibrated from Model 2. The difference between the values (measured–predicted) and the probability at the test sample origin from the geographic assignment are also shown.

Test Sample	δ^2^H_measured_ (‰)	δ^2^H_predicted_ (‰) Model 2	δ^2^H_measured_− δ^2^H_predicted_ (‰) Model 2	Assignment Probability Model 2
1	-67.8	-68.7	0.9	0.97
2	-70.1	-71.4	1.2	0.95
3	-65.7	-66.6	0.9	0.99
4	-66.3	-68.7	2.4	0.77
5	-70.8	-68.5	-2.2	0.88
6	-86.1	-82.8	-3.2	0.72
7	-89.8	-89.8	0.0	0.99
8	-75.0	-80.2	5.2	0.56
9	-95.2	-94.2	-1.0	0.98
10	-95.9	-99.0	3.1	0.84

### Model 3

Another approach, where sufficient data are available, is to directly model the changing patterns in the target biological material. There are a number of modelling approaches reported in the literature that could be applied to do this, including empirical comparisons using linear regression [8], geostatistical modelling of the semivariance [28] or mechanistic modelling [24]. For comparison purposes, in this investigation, the measured δ^2^H values for the blackberry leaves were empirically modelled by multiple linear regression (Model 3). The parameters of the resulting MLR model for measured blackberry leaf δ^2^H values against daily precipitation weighted environmental variables are shown in Table 5.

**Table 5:** Regression parameters for Model 3.

	Estimate	Std. Error	t-value	Pr(>t)
Intercept	-1030	278	-3.703	0.0003
Latitude (°)	2.872e^-5^	3.096e^-6^	9.277	<0.0001
Altitude (m)	-2.473e^-2^	3.327e^-3^	-7.434	<0.0001
Wind Speed (m/s)	1.341	0.326	4.109	<0.0001
Minimum Temperature (°C)	-2.095	0.857	-2.444	0.0161
Maximum Temperature (°C)	1.052	0.630	1.670	0.0977
Mean Sea Level Pressure (hPa)	0.786	0.278	2.823	0.0056
RSE	3.8, 113 d.f.			
r^2^	0.90			

**Fig 6:**
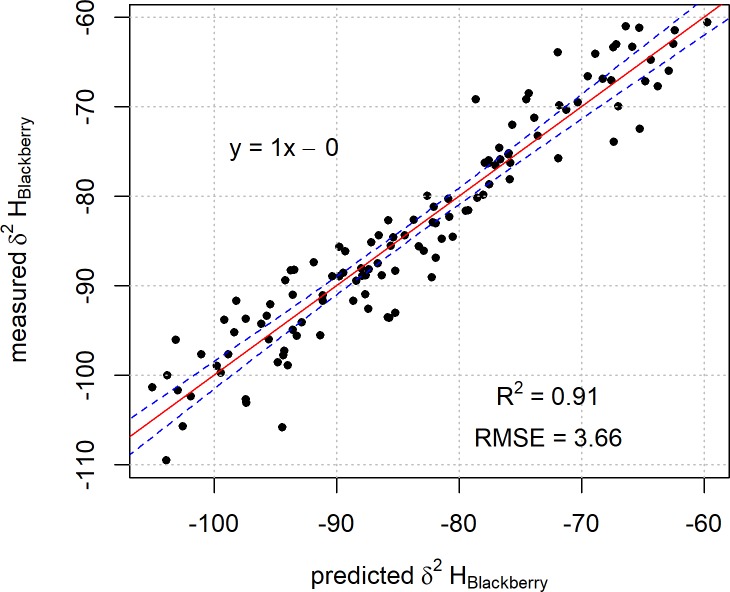
Plot of predicted blackberry leaf δ^2^H values determined from Model 3 against the measured blackberry leaf δ^2^H values. The linear regression line of best fit is represented by the red line with 99% confidence intervals for the fit in blue.

A plot of the regression result from Model 3 against the measured blackberry leaf δ^2^H values is shown in Fig 6.

**Fig 7:**
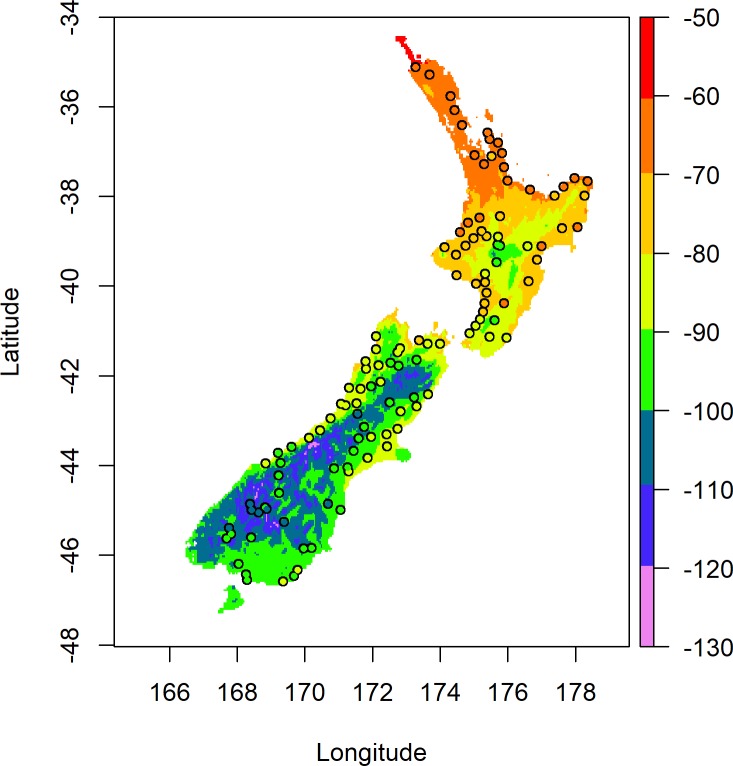
Predicted blackberry leaf δ^2^H values across New Zealand based on Model 3. Coloured points represent the measured δ^2^H values for blackberry leaves sampled from those locations.

In this case, when comparing the δ^2^H values predicted by the model with the measured values, the resultant RMSE (3.7 ‰) is similar to that obtained for Model 2 (4.0 ‰). The isoscape of model-derived predictions for blackberry leaf δ^2^H values based on Model 3 is shown in Fig 7.

A map of the residuals for the model is shown in S3 Fig, which exhibits a random spread across the country. Assessment using a test for Moran’s I was not significant with p = 0.29, similar to that of Model 2, providing little evidence to reject the null hypothesis for the presence of spatial randomness in the residuals. The obvious advantage of empirically modelling the δ^2^H values of the sample material directly is the reduced sources of variance due to removing the need for modelling precipitation δ^2^H and then undertaking further regression. The total variance term used for the geographic assignment of the test set was a summation of the prediction variance of Model 3 determined by cross-validation, which varies spatially, and the residual variance of Model 3. This was determined as 13.5 (s.d. of 3.67 ‰). Given that the predictions were not based on the prediction of precipitation δ^2^H values, there was no need to include the prediction variance and covariance terms associated with this extra modelling step.

The repeated k-folds cross-validation for Model 1 resulted in, at the selected threshold level, 68% of true locations within the determined region of origin. This came with a mean precision of 29 233 km^2^.

The figures showing the likely origin of the test samples are shown in S3 File. 8 out of 10 test samples have a high determined probability at their actual location of origin. The measured δ^2^H values for the test samples and those predicted by Model 3 are shown in Table 6 along with the difference between the two values (measured–predicted) as well as the scaled probability from geographic assignment.

**Table 6:** Measured δ^2^H values for each of the test set blackberry leaf samples and the predicted values from Model 3. The difference between the values (measured–predicted) and the probability at the test sample origin from the geographic assignment are also shown.

Test Sample	δ^2^H_measured_ (‰)	δ^2^H_predicted_ (‰) Model 3	δ^2^H_measured_− δ^2^H_predicted_ (‰) Model 3	Assignment Probability Model 3
1	-67.8	-69.1	1.3	0.94
2	-70.1	-70.0	-0.1	0.99
3	-65.7	-64.4	-1.3	0.94
4	-66.3	-69.9	3.6	0.63
5	-70.8	-70.8	0.0	0.99
6	-86.1	-84.1	-2.0	0.86
7	-89.8	-88.9	-0.9	0.97
8	-75.0	-80.1	5.1	0.39
9	-95.2	-94.5	-0.7	0.98
10	-95.9	-97.3	1.4	0.93

### Model comparisons

Model 3 was compared to the two models (Models 1 and 2), which were calibrated against the different precipitation isoscapes. Validation of the different blackberry leaf δ^2^HHHH isoscape models was undertaken using repeated k-folds cross-validation as well as the external test set (n = 10), which was segregated prior to the development of the models with the training set data. A comparison of the models was undertaken in terms of their individual accuracy and precision utilising the metrics outlined by Vander Zanden et al. [51].

When comparing the cross-validation results of Models 1 and 2, the individual level assignment accuracy was observed to be slightly lower on average for Model 1. A two-sided paired t-test showed the observed difference in accuracy was not significant with p = 0.03 > α = 0.01 (mean = -0.02, t = -2.13, df = 1 199). A similar result was obtained when comparing Models 1 and 3, the accuracy of Model 1 was observed to be slightly lower however this was shown to not be significant with p = 0.35 > α = 0.01 (mean = -0.01, t = -0.93, df = 1 199). Very little difference in accuracy was determined comparing Models 2 and 3 with p = 0.05 > α = 0.01 (mean = -0.00002, t = 1.9243, df = 1 199). This showed that overall there was no real difference in accuracy across the three models.

The individual-level assignment precision was also undertaken through assessment of the change in assignment area from one model to another in thousands of km^2^. Comparison of Models 1 and 2 showed an average reduction in assignment area of 43 349 km^2^ with Model 2 being the more precise (p < 0.01, t = 22.78, df = 1 199). Comparison of Models 1 and 3 gave a similar result with Model 3 being more precise with an average reduction in assignment area of 45 119 km^2^ (p <0.01, t = 22.41, df = 1 199) over Model 1. Comparison of Models 2 and 3 showed a smaller change in precision with Model 3 having an average reduction in assignment area of only 1 769 km^2^ (p < 0.01, t = 3.72, df = 1 199). While this is still a significant change in precision based on the two-sided paired t-test, the smaller average change in assignment area shows that Models 2 and 3 have a more similar precision to each other than Model 1 has to either of the other two models.

The similarity indices for the cross-validation assignments of Model 1 compared to Model 2 ranged from 0.2 proportion of overlap to 0.6, with a mean proportion of overlap of 0.46. For Model 1 compared to Model 3, the similarity indices had a similar range from 0.2 proportion of overlap to 0.55 with a mean proportion of overlap of 0.41. Given that there was no significant difference in accuracy between Model 3 and Model 2 and a small though significant change in precision, it is expected that Model 2 and Model 3 will have similar proportions of overlap in assignment area. This is reflected in the similarity indices of the comparison of Model 2 to Model 3 which ranged from 0.6 proportion of overlap to 0.85, with a mean proportion of overlap of 0.76. Histograms of the change in relative probability (accuracy), change in area (precision) and similarity indices for each of the model comparisons are provided in S4 File.

When comparing the geographic assignment outcomes of the external test set, it was observed that for test sample 8, Model 1, makes predictions which are closer to the measured value than the predictions of Model 2 and Model 3. Test Sample 8 surprisingly originates from the West Coast area of the South Island of New Zealand for which Model 1 did not behave linearly. Further investigation of this test sample shows it exhibits a higher δ^2^H value (-75.0 ‰) than the surrounding training samples measured from the same region (~-85 –-95 ‰). While Model 1 gives a better estimate of the mean δ^2^H value at the location of test sample 8 the efficacy of the geographic assignment is more relevant to food authentication or forensic application as not only the accuracy of the estimation and assignment is important but the precision also. If the intended purpose of the model is to provide a likely area of origin for further investigation, then applying resources across a smaller area is more efficient.

The results of the comparison using the external test set are shown in Table 7; the precision difference is shown as the area difference in thousands of km^2^.

**Table 7:** Pairwise comparisons of accuracy (change in relative probability) and precision (change in area (× 10^3^ km^2^)) of the different isoscape models for each of the ten external test samples. The overall mean accuracy and precision are also shown along with calculated p-values from two-sided paired t-tests (d.f. = 9 in each instance).

Test Sample	Accuracy difference Model 1 -Model 2	Precision difference Model 1 -Model 2	Accuracy difference Model 1—Model 3	Precision difference Model 1 -Model 3	Accuracy difference Model 2 -Model 3	Precision difference Model 2 -Model 3
1	-0.35	26.39	-0.31	24.47	0.03	-1.92
2	-0.13	17.48	-0.18	26.06	-0.05	8.58
3	-0.27	7.83	0.22	-21.37	0.05	-29.20
4	-0.35	26.67	-0.21	30.09	0.14	3.42
5	-0.05	15.35	-0.16	21.97	-0.11	6.62
6	0.22	24.41	0.09	35.90	-0.14	11.48
7	-0.06	55.28	-0.03	55.01	0.03	-0.27
8	0.43	-25.42	0.60	-44.18	0.17	-18.77
9	-0.01	22.22	-0.01	28.02	0.00	5.80
10	-0.08	56.26	-0.17	70.05	-0.09	13.79
Mean Difference	-0.06	22.65	-0.06	24.28	0.003	1.631
p-value (t-test)	0.43	0.01	0.48	0.02	0.91	0.61

While individual differences in accuracy can be seen across the external test sample assignments, the different models show no significant change in mean accuracy (p-values >0.1) when they are compared, as observed from cross-validation. However, the mean precision of the assignments made by the isoscape based on Model 1 is significantly worse than the precision of the assignments made by the isoscapes based on Model 2 (p = 0.01) and Model 3 (p = 0.02). The difference in the precision of assignments made between the isoscapes based on Model 2 and Model 3 was not significant (p = 0.61) with Model 3 having an average 1 631 km^2^ smaller assignment area than Model 2 across all test samples. This is in agreement with the assessments of the accuracy and precision determined by repeated k-folds cross-validation.

Table 8 shows the calculated similarity indices for the 10 test samples between each of the considered isoscapes.

**Table 8:** Similarity indices comparing the proportion of overlap between the different assignment maps for each of the 10 test samples.

Test Sample	Similarity Rogers-Baisden	Similarity Rogers-Direct	Similarity Baisden-Direct
1	0.46	0.43	0.84
2	0.46	0.42	0.82
3	0.40	0.39	0.86
4	0.42	0.40	0.86
5	0.45	0.42	0.82
6	0.34	0.34	0.83
7	0.39	0.38	0.83
8	0.42	0.41	0.85
9	0.53	0.48	0.86
10	0.53	0.49	0.85

The similarity indices show the assignment areas from the isoscape based on Model 1 for each test sample are not very similar to those based on Model 2 or Model 3; with mean proportions of overlap of 0.44 and 0.42 respectively. Model 2 and Model 3 show a higher degree of similarity with a mean proportion of overlap of 0.84. These outcomes are in line with those from the repeated k-folds cross validation.

## Conclusions

Two previously reported precipitation isoscapes were calibrated to the δ^2^H measurements of 120 samples of blackberry leaves from across New Zealand (Models 1 and 2). The measured δ^2^H values of the 120 blackberry leaf samples were also modelled against daily weighted environmental variables (Model 3). The three resulting isoscapes were compared to determine the more appropriate for geographic assignment. Of the three isoscapes, Model 1 which was based on the precipitation isoscape reported by Rogers et al. [14] was the least precise in terms of geographic assignment and exhibited non-linear behaviour in the underlying calibration from precipitation δ^2^H values to blackberry leaf δ^2^H values. This resulted in a skewed prediction surface, higher residual error and higher prediction error which gave rise to the lack of relative precision. The non-linear behaviour was surmised to be due to the incorporation of long-term climate information, which was inappropriate for the shorter integration time of δ^2^H into blackberry leaves. Model 2, which was based on the precipitation isoscape reported by Baisden et al. [11] performed with high accuracy when making assignments of the external test set, including 9 out of 10 true locations within the assignment area. The model also had significantly higher precision when compared to the result of Model 1 based on the undertaken cross-validation and external test set. The precision of Model 2 was not significantly different from the precision of Model 3. The change in precision between Model 2 and Model 3 was considered significant based on the cross-validation result, however. the difference in average area between the two models was approximately 26 times smaller than the average differences between these models and Model 1. Model 3 involved modelling of climate variables to the δ^2^H values of the blackberry to determine an isoscape for geographic assignment. However, the approach taken to determine Model 3 requires a large number of measurements of the plant material from representative sites across the area of interest to capture adequate variation. This may be difficult for plant materials of a more clandestine nature. Given the agreement in accuracy, precision and the high degree of similarity between Model 3 and Model 2; the approach of calibrating the precipitation isoscape reported by Baisden et al [11] is as viable as modelling plant material δ^2^H values against the climate parameters. The approach for determining the precipitation δ^2^H isoscape reported by Baisden et al. [11] is flexible enough that it can represent a range of integration times for the plant material. It appears to be an appropriate choice of underlying model against which specific plant material δ^2^H values could be calibrated for New Zealand and could be utilised as the starting point to calibrate multiple different plant materials against.

## Supporting information

S1 Table The measured δ^2^H values of the 130 blackberry leaf samples. The training set consists of 120 samples with geographic coordinates in decimal degrees (WGS84). The test set consists of 10 samples with geographic coordinates in decimal degrees (WGS84).
(XLSX)

S2 Table The New Zealand CDRP isotopes in precipitation data used for determining precipitation isoscapes for New Zealand. The data set consists of monthly δ^2^H and δ^18^O measurements from August 2007 to December 2009 for 51 sites with geographic coordinates in decimal degrees (WGS84).
(XLSX)

S3 Table R packages utilised for this investigation and their accompanying citations. (XLSX)

S1 Fig Map of the residuals from the linear calibration of δ^2^H values in precipitation from Rogers et al. to δ^2^H values in blackberry leaf (Model 1). (TIF)

S2 Fig Map of the residuals from the linear calibration of δ^2^H values in precipitation from Baisden et al. to δ^2^H values in blackberry leaf (Model 2). (TIF)

S3 Fig Map of the residuals from the multiple linear regression of δ^2^H values in blackberry leaf to daily precipitation-weighted environmental variables obtained from the NIWA VCSN database (Model 3). (TIF)

S1 File Geographic assignment maps for test samples 1 to 10 using the δ^2^H blackberry leaf isoscape determined by calibration of the δ^2^H precipitation isoscape reported by Rogers et al. (Model 1). Likelihood of true origin is scaled from 0 (black) to 1 (green).
(PDF)

S2 File Geographic assignment maps for test samples 1 to 10 using the δ^2^H blackberry leaf isoscape determined by calibration of the δ^2^H precipitation isoscape reported by Baisden et al. (Model 2). Likelihood of true origin is scaled from 0 (black) to 1 (green).
(PDF)

S3 File Geographic assignment maps for test samples 1 to 10 using the δ^2^H blackberry leaf isoscape determined by direct modelling of daily precipitation-weighted environmental variables obtained from the NIWA VCSN database (Model 3). Likelihood of true origin is scaled from 0 (black) to 1 (green).
(PDF)

S4 File Histograms of the change in relative probability (accuracy), the change in area (precision) and similarity indices for each of the model comparisons based on repeated k-folds cross-validation. Red lines indicate the mean value of each comparison.
(PDF)

## References

[1] Bowen GJ. Isoscapes: Spatial Pattern in Isotopic Biogeochemistry. Annual Review of Earth and Planetary Sciences. 2010;38: 161–187. 10.1146/annurev-earth-040809-152429

[2] West JB, Bowen GJ, Ehleringer JR. Predicting Hydrogen and Oxygen Stable Isotope Ratios of Plants Across Terrestrial Surfaces: Plant IsoScapes. AGU Fall Meeting Abstracts. 2005;22: B22B–07.

[3] Araguás‐Araguás L, Froehlich K, Rozanski K. Deuterium and oxygen-18 isotope composition of precipitation and atmospheric moisture. Hydrological Processes. 2000;14: 1341–1355. 10.1002/1099-1085(20000615)14:8<1341::AID-HYP983>3.0.CO;2-Z

[4] IAEA/WMO. Global Network of Isotopes in Precipitation. The GNIP Database. 2019 [cited 10 Aug 2019]. Available: https://nucleus.iaea.org/wiser

[5] Terzer S, Wassenaar LI, Araguás-Araguás LJ, Aggarwal PK. Global isoscapes for δ18O and δ2H in precipitation: improved prediction using regionalized climatic regression models. Hydrology and Earth System Sciences. 2013;17: 4713–4728. 10.5194/hess-17-4713-2013

[6] Bowen GJ, Revenaugh J. Interpolating the isotopic composition of modern meteoric precipitation: ISOTOPIC COMPOSITION OF MODERN PRECIPITATION. Water Resources Research. 2003;39 10.1029/2003WR002086

[7] Bowen GJ, Wilkinson B. Spatial distribution of δ18O in meteoric precipitation. Geology. 2002;30: 315–318. 10.1130/0091-7613(2002)030<0315:SDOOIM>2.0.CO;2

[8] van der Veer G, Voerkelius S, Lorentz G, Heiss G, Hoogewerff JA. Spatial interpolation of the deuterium and oxygen-18 composition of global precipitation using temperature as ancillary variable. Journal of Geochemical Exploration. 2009;101: 175–184. 10.1016/j.gexplo.2008.06.008

[9] Jouzel J, Hoffmann G, Koster RD, Masson V. Water isotopes in precipitation:: data/model comparison for present-day and past climates. Quaternary Science Reviews. 2000;19: 363–379. 10.1016/S0277-3791(99)00069-4

[10] Mathieu R, Pollard D, Cole JE, White JWC, Webb RS, Thompson SL. Simulation of stable water isotope variations by the GENESIS GCM for modern conditions. Journal of Geophysical Research: Atmospheres. 2002;107: ACL 2-1–ACL 2–18. 10.1029/2001JD900255

[11] Baisden WT, Keller ED, Hale RV, Frew RD, Wassenaar LI. Precipitation isoscapes for New Zealand: enhanced temporal detail using precipitation-weighted daily climatology. Isotopes in Environmental and Health Studies. 2016;52: 343–352. 10.1080/10256016.2016.1153472 27007914

[12] Frew Russell, Robert Van Hale Tony Moore, Darling Michael. A stable isotope rainfall map for the protection of New Zealand’s biological and environmental resources. 2011 p. 57.

[13] Ehtesham E, Baisden WT, Keller ED, Hayman AR, Van Hale R, Frew RD. Correlation between precipitation and geographical location of the δ2H values of the fatty acids in milk and bulk milk powder. Geochimica et Cosmochimica Acta. 2013;111: 105–116. 10.1016/j.gca.2012.10.026

[14] Rogers KM, Wassenaar LI, Soto DX, Bartle JA. A feather-precipitation hydrogen isoscape model for New Zealand: implications for eco-forensics. Ecosphere. 2012;3: art62 10.1890/ES11-00343.1

[15] Bowen GJ, Liu Z, Vander Zanden HB, Zhao L, Takahashi G. Geographic assignment with stable isotopes in IsoMAP. Kurle C, editor. Methods Ecol Evol. 2014;5: 201–206. 10.1111/2041-210X.12147

[16] Wunder MB, Norris DR. Chapter 8—Design and Analysis for Isotope-Based Studies of Migratory Animals In: Hobson KA, Wassenaar LI, editors. Tracking Animal Migration with Stable Isotopes (Second Edition). Academic Press; 2019 pp. 191–206. 10.1016/B978-0-12-814723-8.00008-8

[17] Vander Zanden HB, Nelson DM, Wunder MB, Conkling TJ, Katzner T. Application of isoscapes to determine geographic origin of terrestrial wildlife for conservation and management. Biological Conservation. 2018;228: 268–280. 10.1016/j.biocon.2018.10.019

[18] Hren MT, Pagani M, Erwin DM, Brandon M. Biomarker reconstruction of the early Eocene paleotopography and paleoclimate of the northern Sierra Nevada. Geology. 2010;38: 7–10. 10.1130/G30215.1

[19] Sachse D, Billault I, Bowen GJ, Chikaraishi Y, Dawson TE, Feakins SJ, et al Molecular Paleohydrology: Interpreting the Hydrogen-Isotopic Composition of Lipid Biomarkers from Photosynthesizing Organisms. Annu Rev Earth Planet Sci. 2012;40: 221–249. 10.1146/annurev-earth-042711-105535

[20] Sachse D, Radke J, Gleixner G. Hydrogen isotope ratios of recent lacustrine sedimentary n-alkanes record modern climate variability. Geochimica et Cosmochimica Acta. 2004;68: 4877–4889. 10.1016/j.gca.2004.06.004

[21] Zhuang G, Brandon MT, Pagani M, Krishnan S. Leaf wax stable isotopes from Northern Tibetan Plateau: Implications for uplift and climate since 15 Ma. Earth and Planetary Science Letters. 2014;390: 186–198. 10.1016/j.epsl.2014.01.003

[22] Brett MJ, Baldini JUL, Gröcke DR. Environmental controls on stable isotope ratios in New Zealand Podocarpaceae: Implications for palaeoclimate reconstruction. Global and Planetary Change. 2014;120: 38–45. 10.1016/j.gloplacha.2014.05.010

[23] Hou J, D’Andrea WJ, Huang Y. Can sedimentary leaf waxes record D/H ratios of continental precipitation? Field, model, and experimental assessments. Geochimica et Cosmochimica Acta. 2008;72: 3503–3517. 10.1016/j.gca.2008.04.030

[24] West JB, Kreuzer HW, Ehleringer JR. Approaches to Plant Hydrogen and Oxygen Isoscapes Generation In: West JB, Bowen GJ, Dawson TE, Tu KP, editors. Isoscapes: Understanding movement, pattern, and process on Earth through isotope mapping. Dordrecht: Springer Netherlands; 2010 pp. 161–178. 10.1007/978-90-481-3354-3_8

[25] Roden JS, Lin G, Ehleringer JR. A mechanistic model for interpretation of hydrogen and oxygen isotope ratios in tree-ring cellulose. Geochimica et Cosmochimica Acta. 2000;64: 21–35. 10.1016/S0016-7037(99)00195-7

[26] Stern B, Moore CDL, Heron C, Pollard AM. Bulk Stable Light Isotopic Ratios in Recent and Archaeological Resins: Towards Detecting the Transport of Resins in Antiquity?*. Archaeometry. 2008;50: 351–370. 10.1111/j.1475-4754.2007.00357.x

[27] Kelly S, Heaton K, Hoogewerff J. Tracing the geographical origin of food: The application of multi-element and multi-isotope analysis. Trends in Food Science & Technology. 2005;16: 555–567. 10.1016/j.tifs.2005.08.008

[28] Gori Y, Stradiotti A, Camin F. Timber isoscapes. A case study in a mountain area in the Italian Alps. Heinze B, editor. PLoS ONE. 2018;13: e0192970 10.1371/journal.pone.0192970 29451907 PMC5815615

[29] Booth AL, Wooller MJ, Howe T, Haubenstock N. Tracing geographic and temporal trafficking patterns for marijuana in Alaska using stable isotopes (C, N, O and H). Forensic Science International. 2010;202: 45–53. 10.1016/j.forsciint.2010.04.025 20494534

[30] Hurley JM, West JB, Ehleringer JR. Stable isotope models to predict geographic origin and cultivation conditions of marijuana. Science & Justice. 2010;50: 86–93. 10.1016/j.scijus.2009.11.003 20470741

[31] Cerling TE, Barnette JE, Bowen GJ, Chesson LA, Ehleringer JR, Remien CH, et al Forensic Stable Isotope Biogeochemistry. Annual Review of Earth and Planetary Sciences. 2016;44: 175–206. 10.1146/annurev-earth-060115-012303

[32] Hurley JM, West JB, Ehleringer JR. Tracing retail cannabis in the United States: Geographic origin and cultivation patterns. International Journal of Drug Policy. 2010;21: 222–228. 10.1016/j.drugpo.2009.08.001 19765966

[33] Carter JF, Yates HSA, Tinggi U. A global survey of the stable isotope and chemical compositions of bottled and canned beers as a guide to authenticity. Science & Justice. 2015;55: 18–26. 10.1016/j.scijus.2014.05.002 25577003

[34] Carter JF, Yates HSA, Tinggi U. Stable Isotope and Chemical Compositions of European and Australasian Ciders as a Guide to Authenticity. J Agric Food Chem. 2015;63: 975–982. 10.1021/jf5030054 25536876

[35] Holder PW, Armstrong K, Van Hale R, Millet M-A, Frew R, Clough TJ, et al Isotopes and Trace Elements as Natal Origin Markers of Helicoverpa armigera–An Experimental Model for Biosecurity Pests. Doucet D, editor. PLoS ONE. 2014;9: e92384 10.1371/journal.pone.0092384 24664236 PMC3963883

[36] Holder PW, Frew R, Van Hale R. The Geographic Origin of an Intercepted Biosecurity Pest Beetle Assigned Using Hydrogen Stable Isotopes. J Econ Entomol. 2015;108: 834–837. 10.1093/jee/tou097 26470196

[37] Wunder M. Using Isoscapes to Model Probability Surfaces for Determining Geographic Origins. Isoscapes: Understanding movement, pattern, and process on Earth through isotope mapping. 2010 pp. 251–270. 10.1007/978-90-481-3354-3_12

[38] Davis Mark, Meurk Colin. Protecting and restoring our natural heritage—a practical guide. Dept. of Conservation, New Zealand; 2001.

[39] Maga JA, Squire CK, Hughes HG. Bramble Dried Leaf Volatiles In: Charalambous G, editor. Developments in Food Science. Elsevier; 1992 pp. 145–148. 10.1016/B978-0-444-88834-1.50016-8

[40] Meier‐Augenstein W, Chartrand MMG, Kemp HF, St‐Jean G. An inter-laboratory comparative study into sample preparation for both reproducible and repeatable forensic 2H isotope analysis of human hair by continuous flow isotope ratio mass spectrometry. Rapid Communications in Mass Spectrometry. 2011;25: 3331–3338. 10.1002/rcm.5235 22006397

[41] Qi H, Coplen TB. Investigation of preparation techniques for δ2H analysis of keratin materials and a proposed analytical protocol. Rapid Commun Mass Spectrom. 2011;25: 2209–2222. 10.1002/rcm.5095 21735504

[42] Soto DX, Koehler G, Wassenaar LI, Hobson KA. Re-evaluation of the hydrogen stable isotopic composition of keratin calibration standards for wildlife and forensic science applications. Rapid Commun Mass Spectrom. 2017;31: 1193–1203. 10.1002/rcm.7893 28475227

[43] WorldClim—Global Climate Data | Free climate data for ecological modeling and GIS. [cited 23 Aug 2019]. Available: http://worldclim.org/

[44] Virtual Climate Station data and products. In: NIWA [Internet]. 17 Aug 2012 [cited 23 Aug 2019]. Available: https://www.niwa.co.nz/climate/our-services/virtual-climate-stations

[45] Fick SE, Hijmans RJ. WorldClim 2: new 1-km spatial resolution climate surfaces for global land areas. International Journal of Climatology. 2017;37: 4302–4315. 10.1002/joc.5086

[46] Hijmans RJ, Cameron SE, Parra JL, Jones PG, Jarvis A. Very high resolution interpolated climate surfaces for global land areas. International Journal of Climatology. 2005;25: 1965–1978. 10.1002/joc.1276

[47] Tait A, Henderson R, Turner R, Zheng X. Thin plate smoothing spline interpolation of daily rainfall for New Zealand using a climatological rainfall surface. International Journal of Climatology. 2006;26: 2097–2115. 10.1002/joc.1350

[48] Tait A, Woods R. Spatial Interpolation of Daily Potential Evapotranspiration for New Zealand Using a Spline Model. Journal of Hydrometeorology. 2007;8: 430–438. 10.1175/JHM572.1

[49] Courtiol A, Rousset F, Rohwäder M-S, Soto DX, Lehnert LS, Voigt CC, et al Chapter 9—Isoscape Computation and Inference of Spatial Origins With Mixed Models Using the R package IsoriX In: Hobson KA, Wassenaar LI, editors. Tracking Animal Migration with Stable Isotopes (Second Edition). Academic Press; 2019 pp. 207–236. 10.1016/B978-0-12-814723-8.00009-X

[50] Gareth James DW Trevor Hastie, Robert Tibshirani. An introduction to statistical learning: with applications in R. New York: Springer, [2013] ©2013; 2013. Available: https://search.library.wisc.edu/catalog/9910207152902121

[51] Vander Zanden HB, Wunder MB, Hobson KA, Van Wilgenburg SL, Wassenaar LI, Welker JM, et al Contrasting assignment of migratory organisms to geographic origins using long-term versus year-specific precipitation isotope maps. Kurle C, editor. Methods Ecol Evol. 2014;5: 891–900. 10.1111/2041-210X.12229

[52] Hobson KA, Wunder MB, Van Wilgenburg SL, Clark RG, Wassenaar LI. A Method for Investigating Population Declines of Migratory Birds Using Stable Isotopes: Origins of Harvested Lesser Scaup in North America. Rands S, editor. PLoS ONE. 2009;4: e7915 10.1371/journal.pone.0007915 19946360 PMC2776275

[53] R Core Team. R: A Language and Environment for Statistical Computing. Vienna, Austria: R Foundation for Statistical Computing; 2019 Available: https://www.R-project.org.

