# Supplementary figures and images for: A δ2H Isoscape of blackberry as an example application for determining the geographic origins of plant materials in New Zealand
Source: PLoS One. 2019 Dec 9;14(12):e0226152. doi: 10.1371/journal.pone.0226152 (PMC6901217; doi:10.1371/journal.pone.0226152)

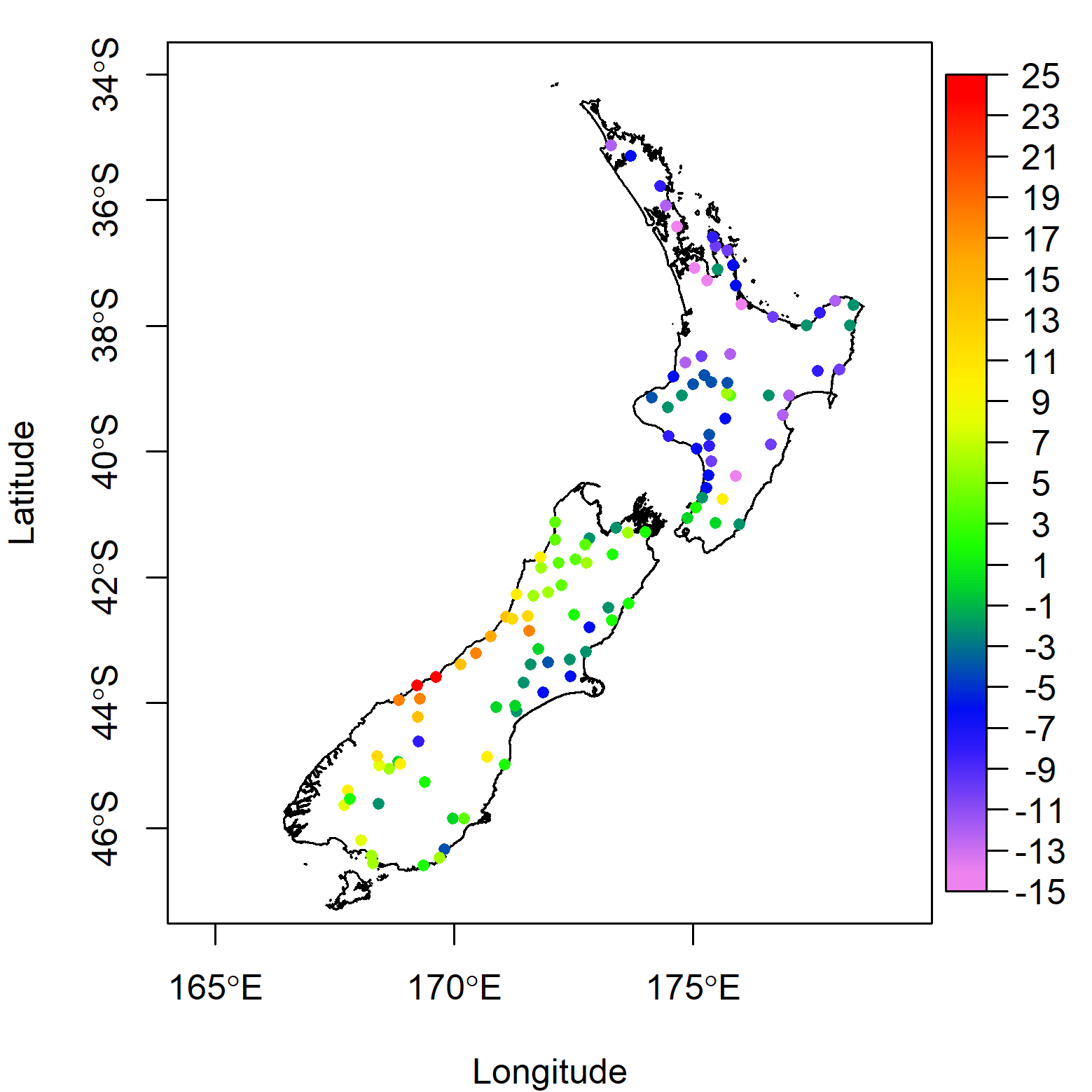

Supplement: S1 Fig — (TIF) [file pone.0226152.s004.tif]

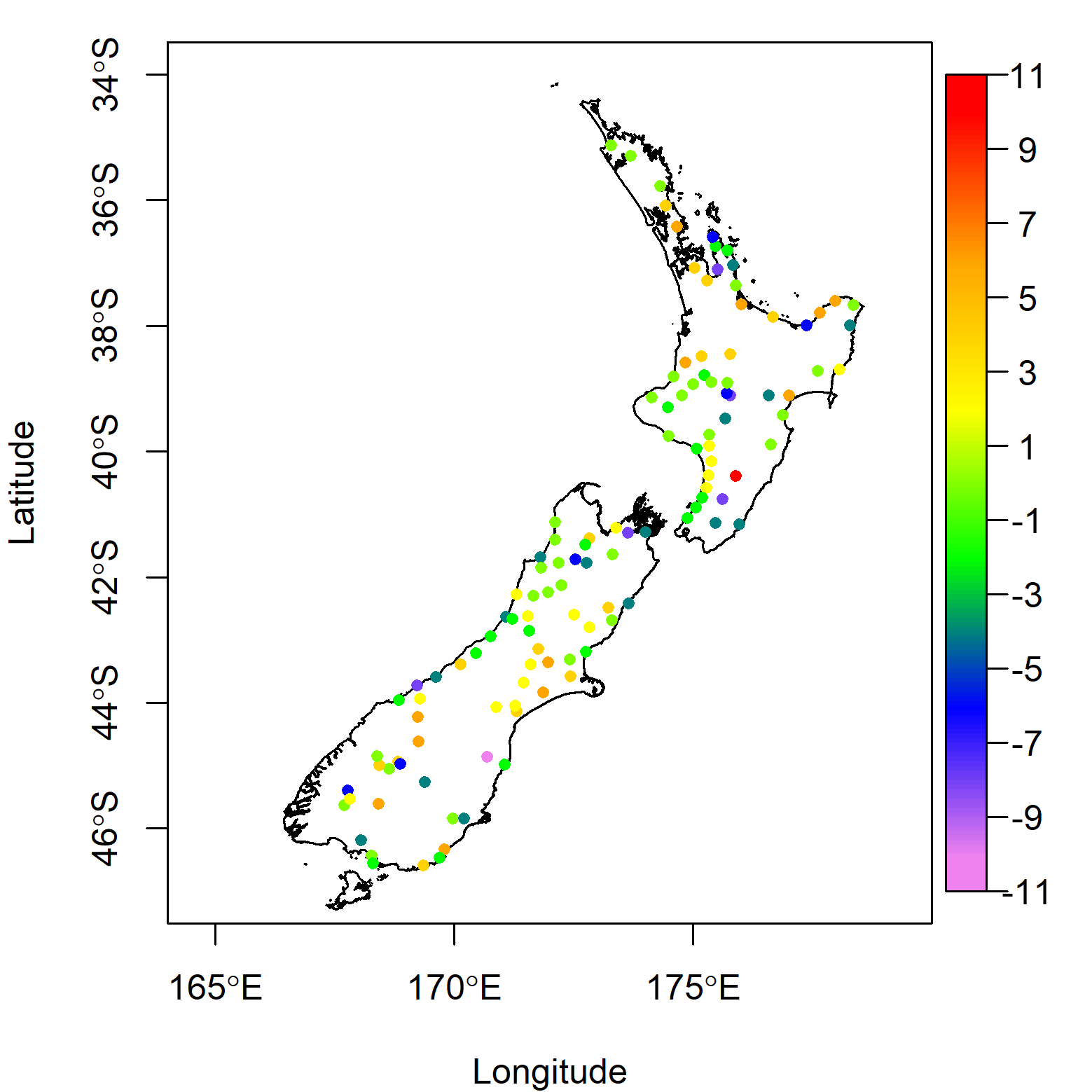

Supplement: S2 Fig — (TIF) [file pone.0226152.s005.tif]

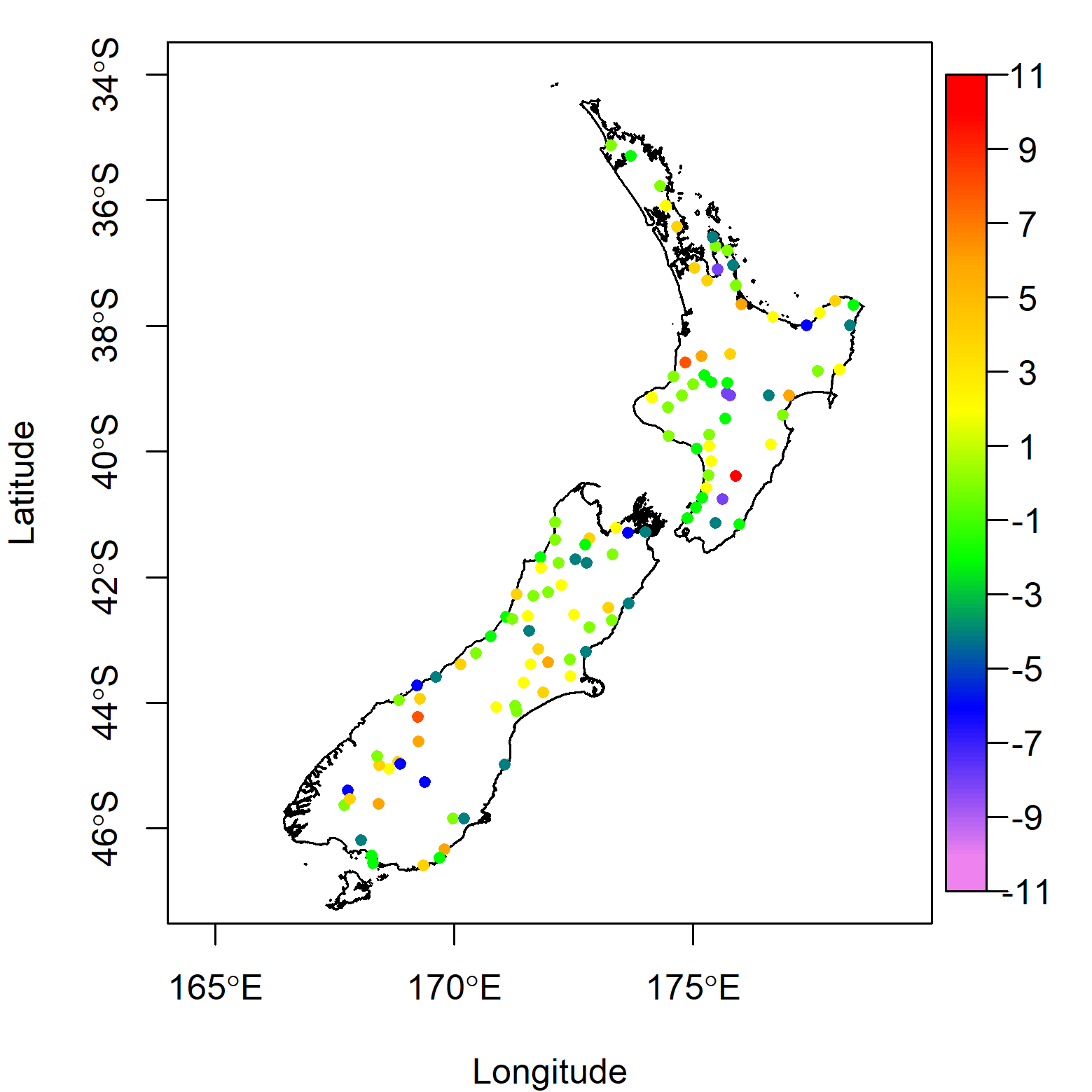

Supplement: S3 Fig — (TIF) [file pone.0226152.s006.tif]
