# Supplementary material for: A δ2H Isoscape of blackberry as an example application for determining the geographic origins of plant materials in New Zealand
Source: PLoS One. 2019 Dec 9;14(12):e0226152. doi: 10.1371/journal.pone.0226152 (PMC6901217; doi:10.1371/journal.pone.0226152)
Supplement: S3 File — Likelihood of true origin is scaled from 0 (black) to 1 (green). (PDF) [file pone.0226152.s009.pdf]

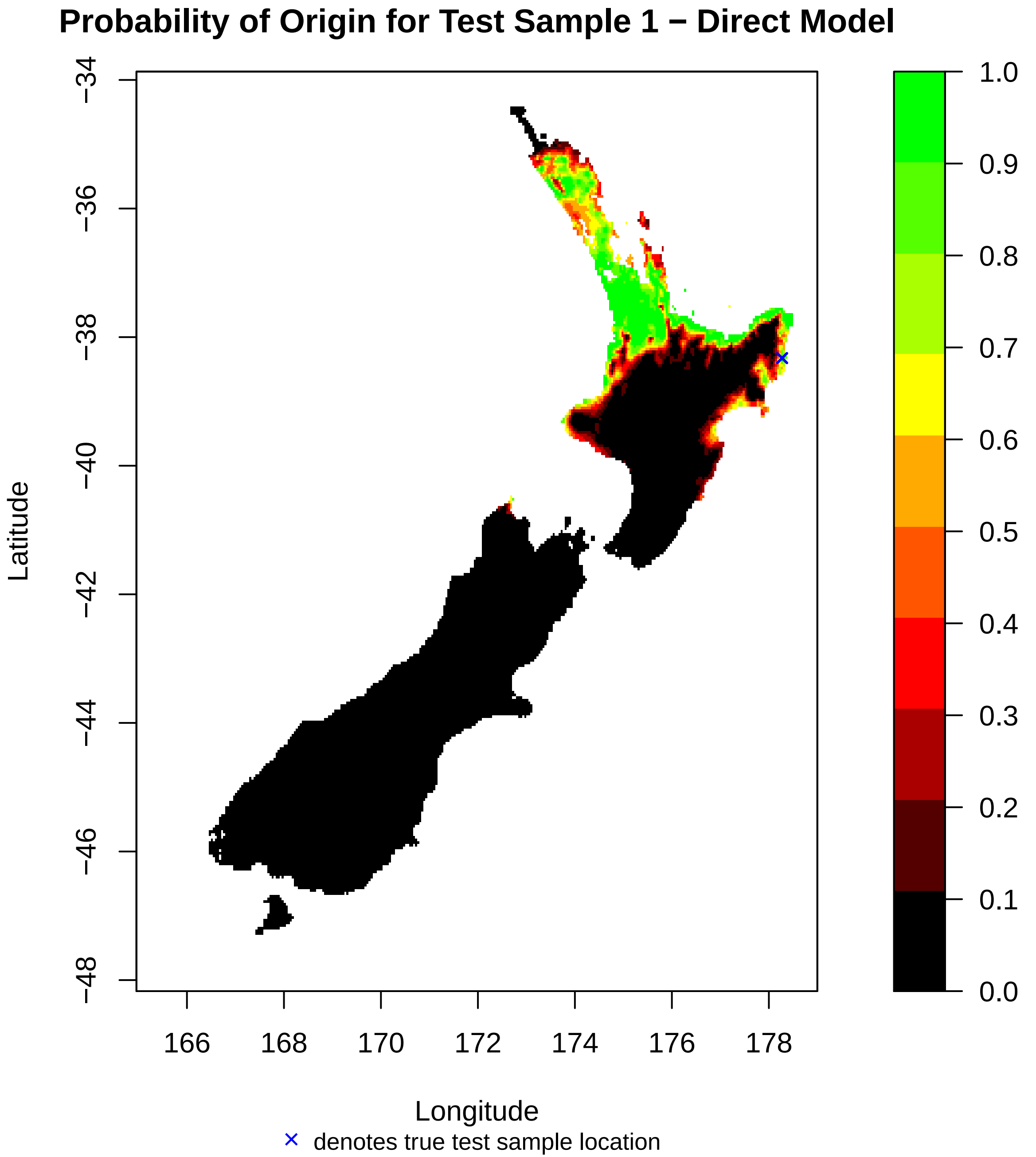

Probability of Origin for Test Sample 1 − Direct Model
Latitude
−34
−36
−38
−40
−42
−44
−46
−48
Longitude
166
168
170
172
174
176
178
1.0
0.9
0.8
0.7
0.6
0.5
0.4
0.3
0.2
0.1
0.0
× denotes true test sample location

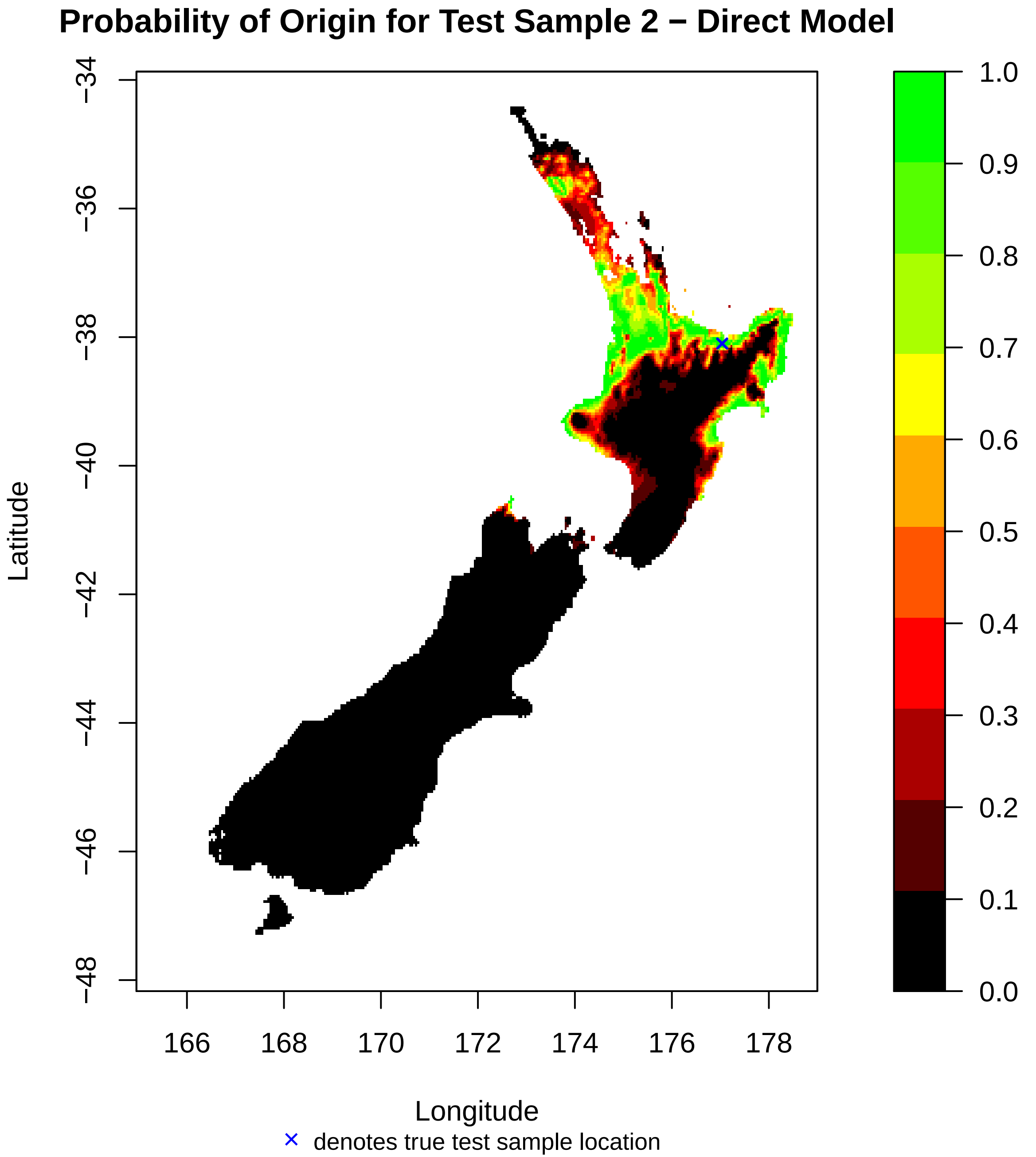
Probability of Origin for Test Sample 2 − Direct Model
Latitude
−34
−36
−38
−40
−42
−44
−46
−48
Longitude
166
168
170
172
174
176
178
1.0
0.9
0.8
0.7
0.6
0.5
0.4
0.3
0.2
0.1
0.0
× denotes true test sample location

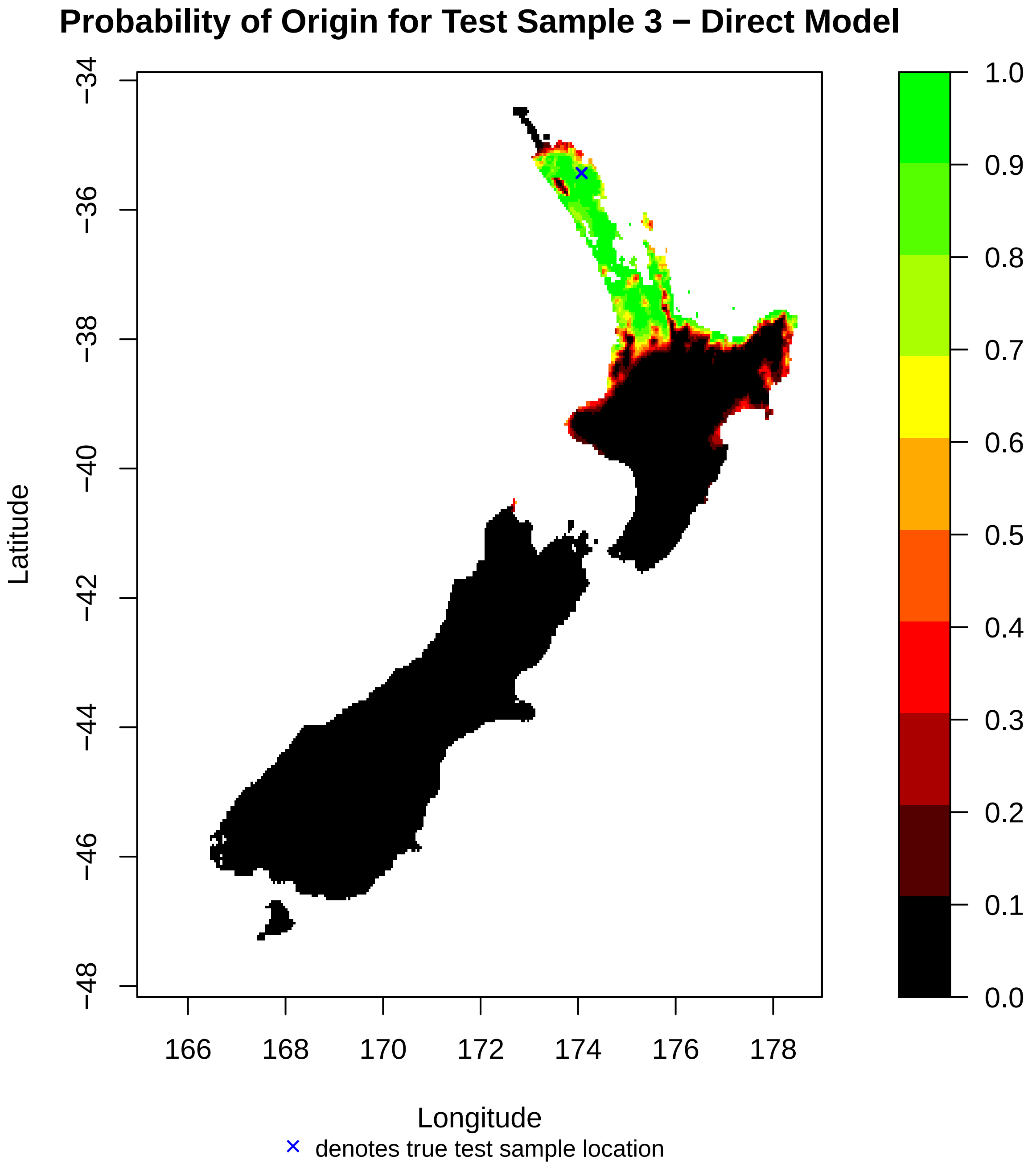
Probability of Origin for Test Sample 3 − Direct Model
Latitude
−34
−36
−38
−40
−42
−44
−46
−48
Longitude
166
168
170
172
174
176
178
1.0
0.9
0.8
0.7
0.6
0.5
0.4
0.3
0.2
0.1
0.0
× denotes true test sample location

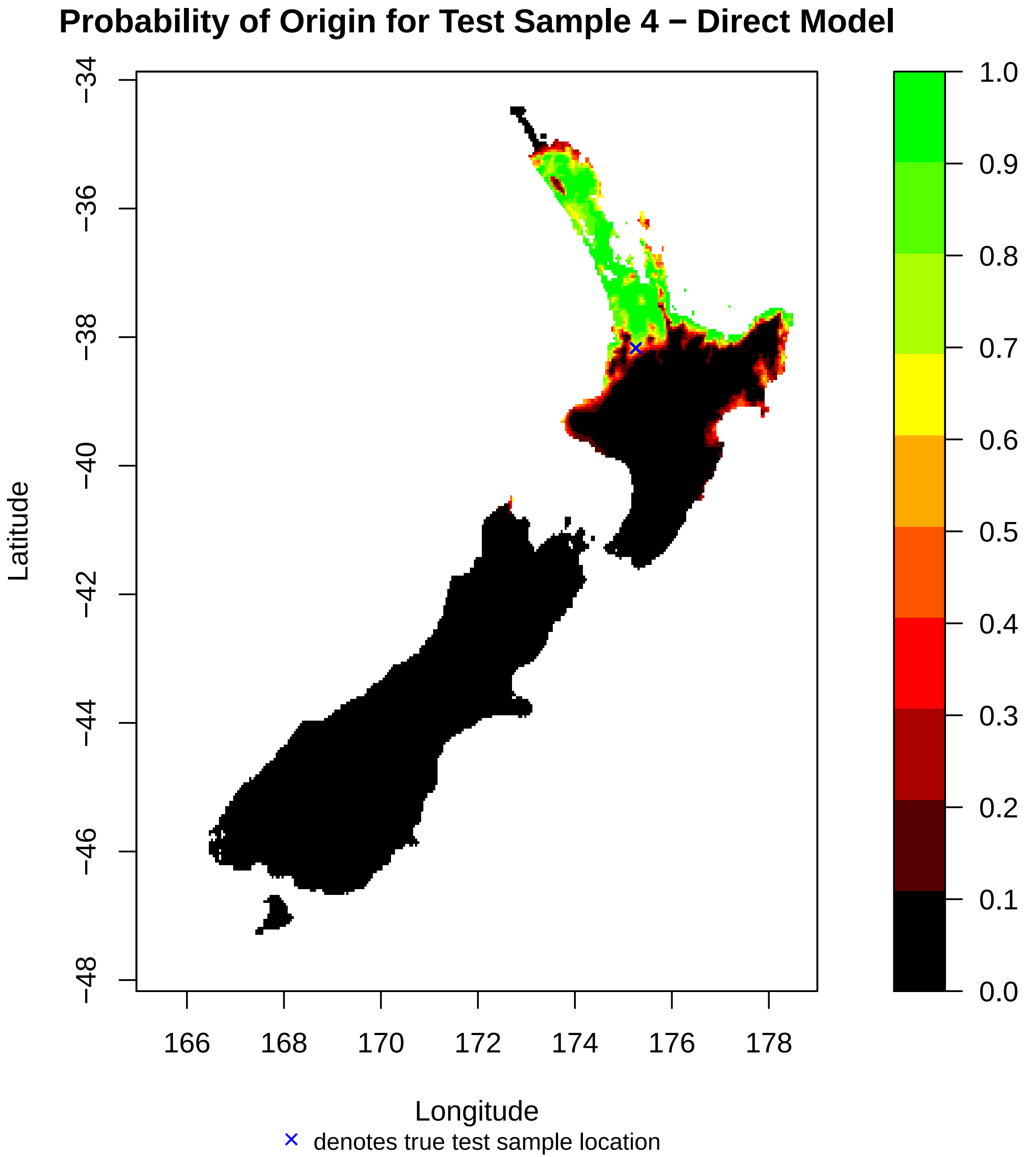
Probability of Origin for Test Sample 4 − Direct Model
Latitude
−34
−36
−38
−40
−42
−44
−46
−48
Longitude
166
168
170
172
174
176
178
1.0
0.9
0.8
0.7
0.6
0.5
0.4
0.3
0.2
0.1
0.0
× denotes true test sample location

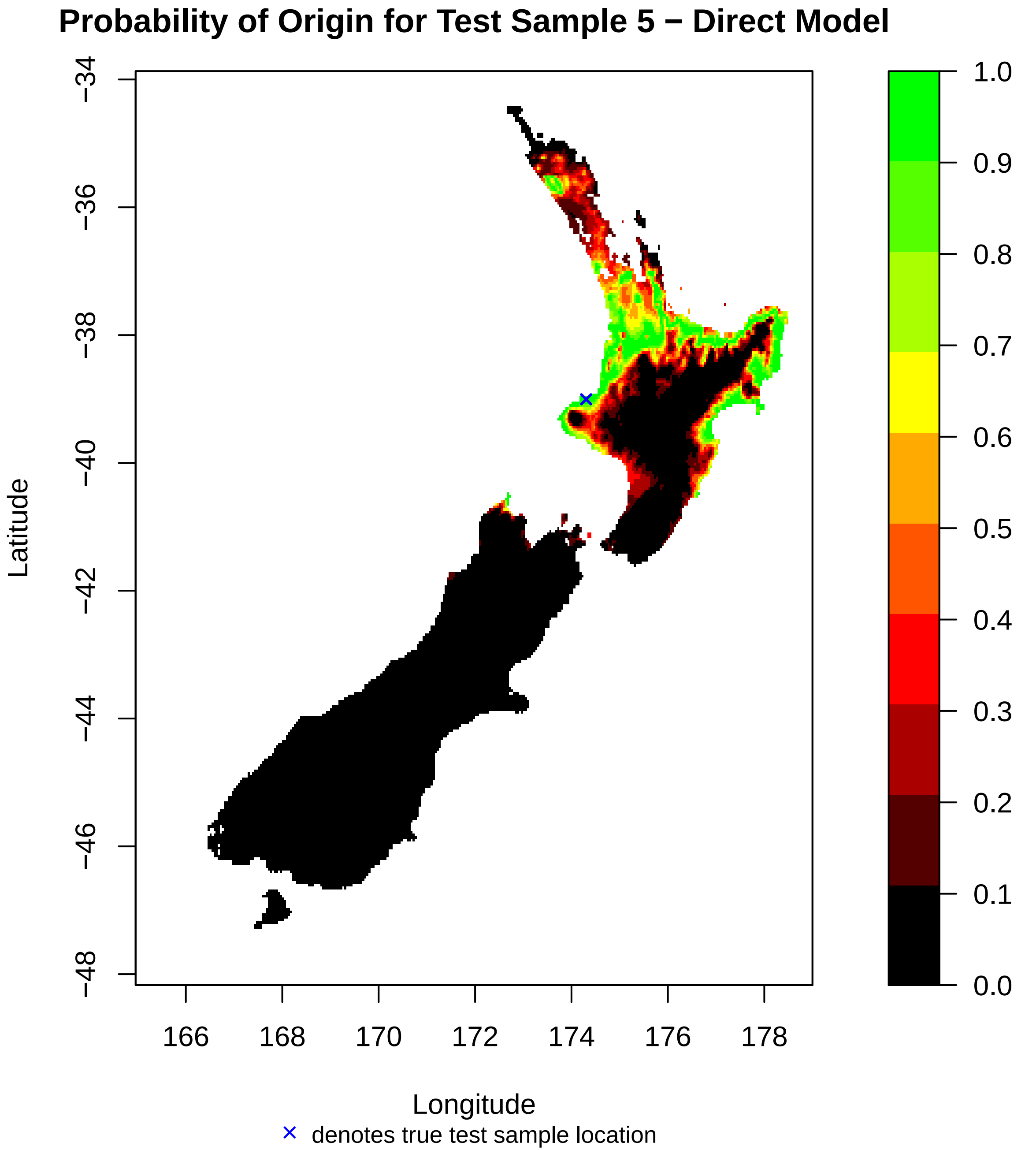
Probability of Origin for Test Sample 5 − Direct Model
Latitude
−34
−36
−38
−40
−42
−44
−46
−48
Longitude
166
168
170
172
174
176
178
1.0
0.9
0.8
0.7
0.6
0.5
0.4
0.3
0.2
0.1
0.0
× denotes true test sample location

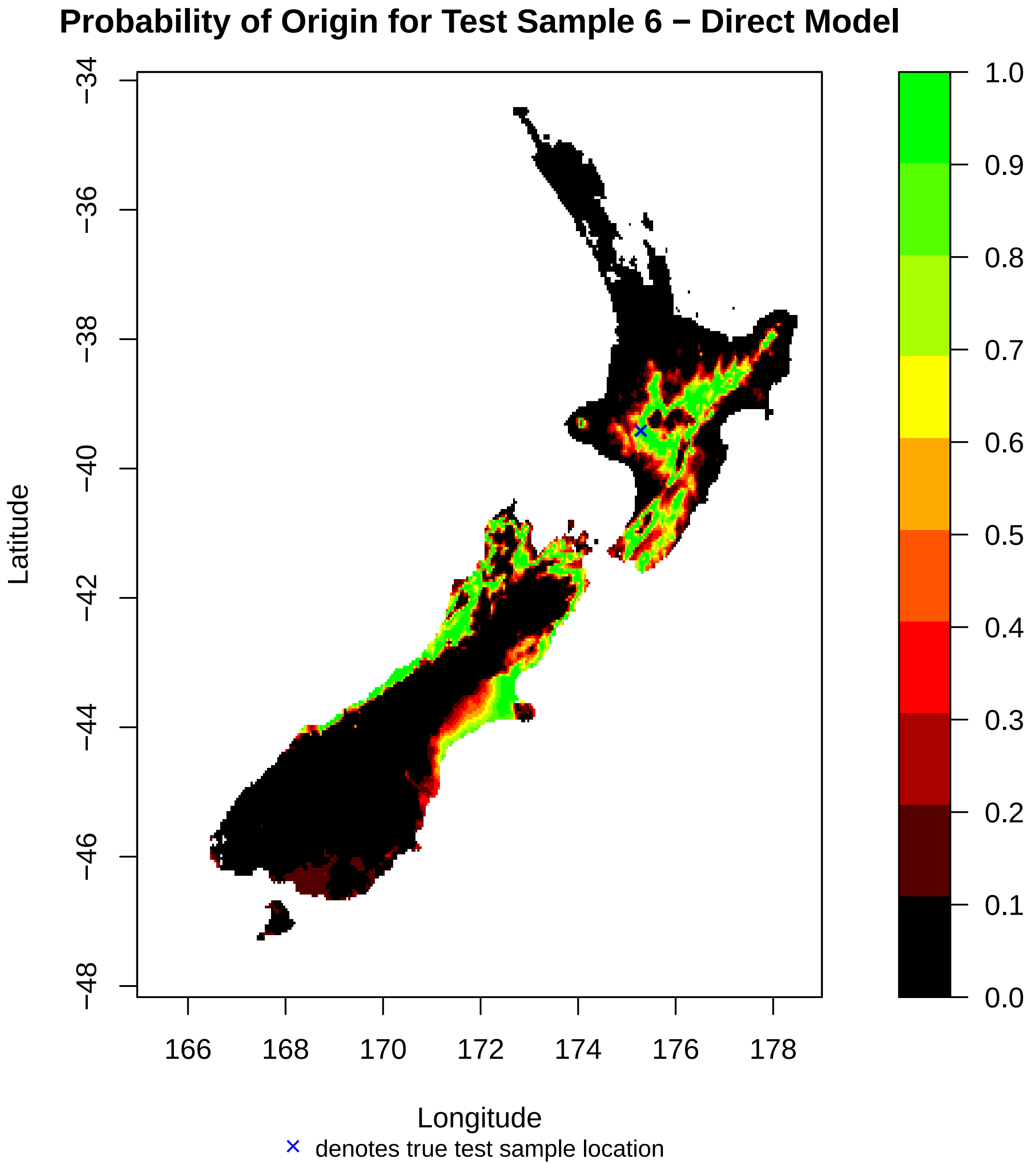
Probability of Origin for Test Sample 6 − Direct Model
Latitude
−34
−36
−38
−40
−42
−44
−46
−48
Longitude
166
168
170
172
174
176
178
1.0
0.9
0.8
0.7
0.6
0.5
0.4
0.3
0.2
0.1
0.0
× denotes true test sample location

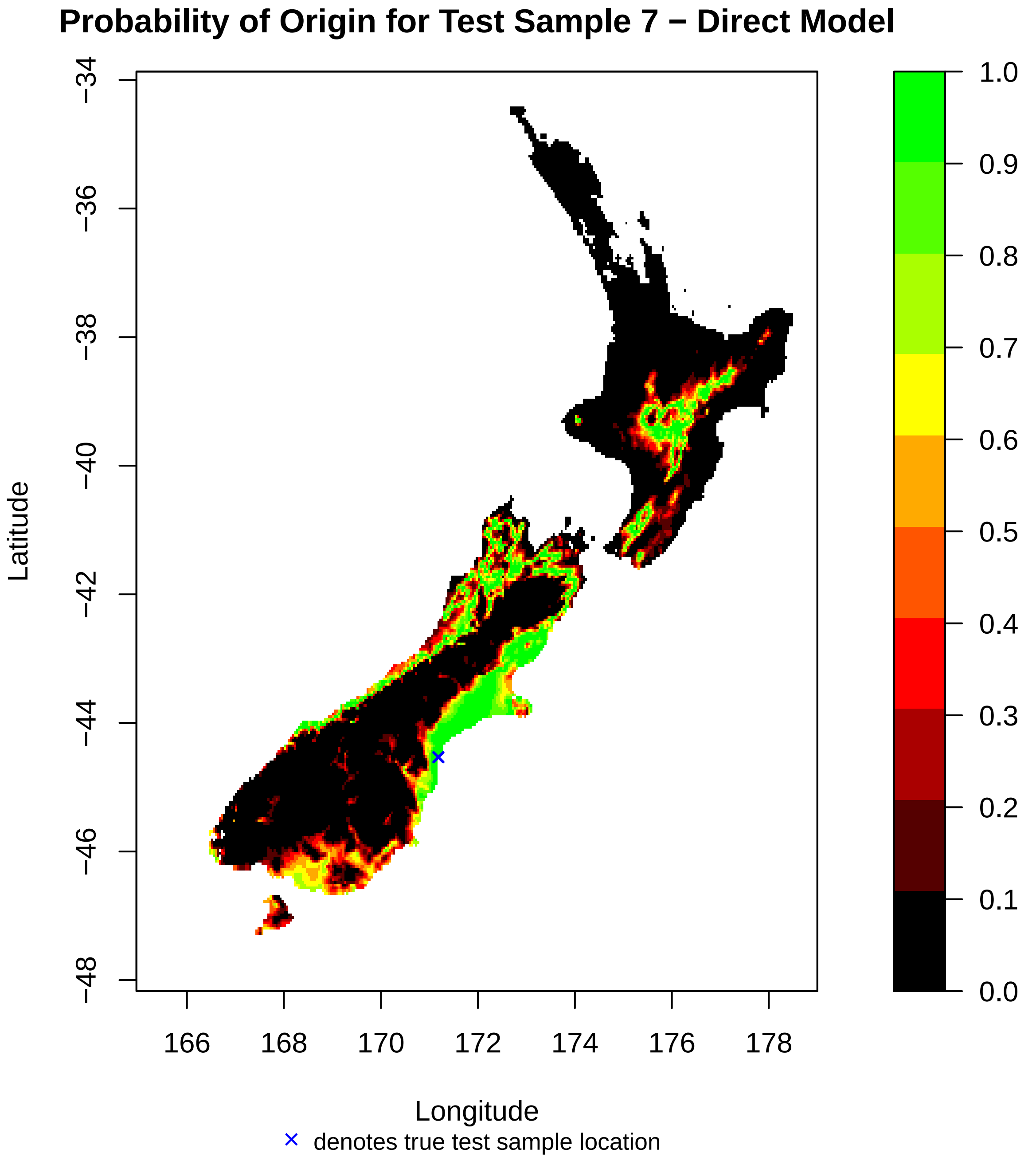
Probability of Origin for Test Sample 7 − Direct Model
Latitude
−34
−36
−38
−40
−42
−44
−46
−48
Longitude
166
168
170
172
174
176
178
1.0
0.9
0.8
0.7
0.6
0.5
0.4
0.3
0.2
0.1
0.0
× denotes true test sample location

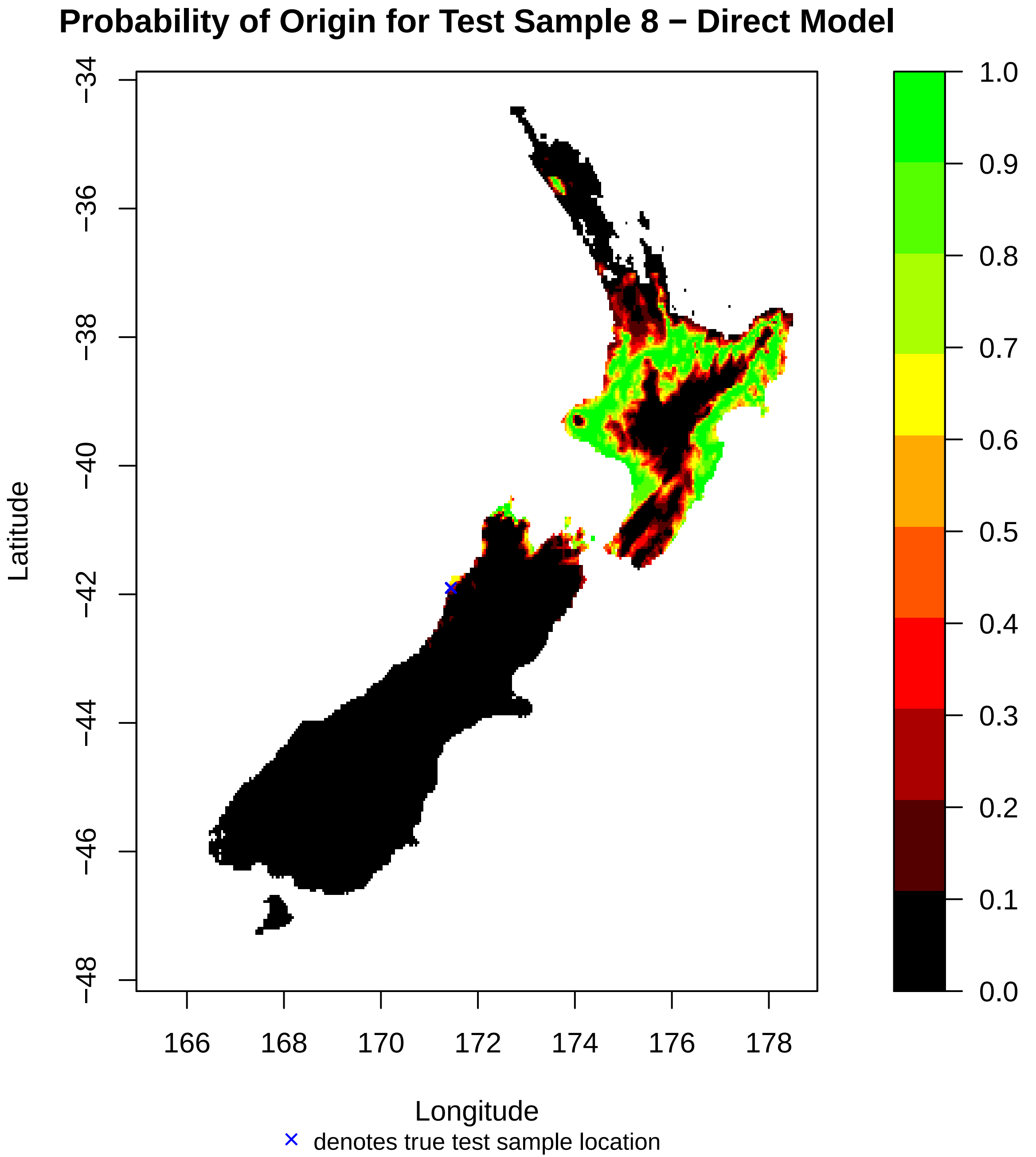
Probability of Origin for Test Sample 8 − Direct Model
Latitude
−34
−36
−38
−40
−42
−44
−46
−48
Longitude
166
168
170
172
174
176
178
1.0
0.9
0.8
0.7
0.6
0.5
0.4
0.3
0.2
0.1
0.0
× denotes true test sample location

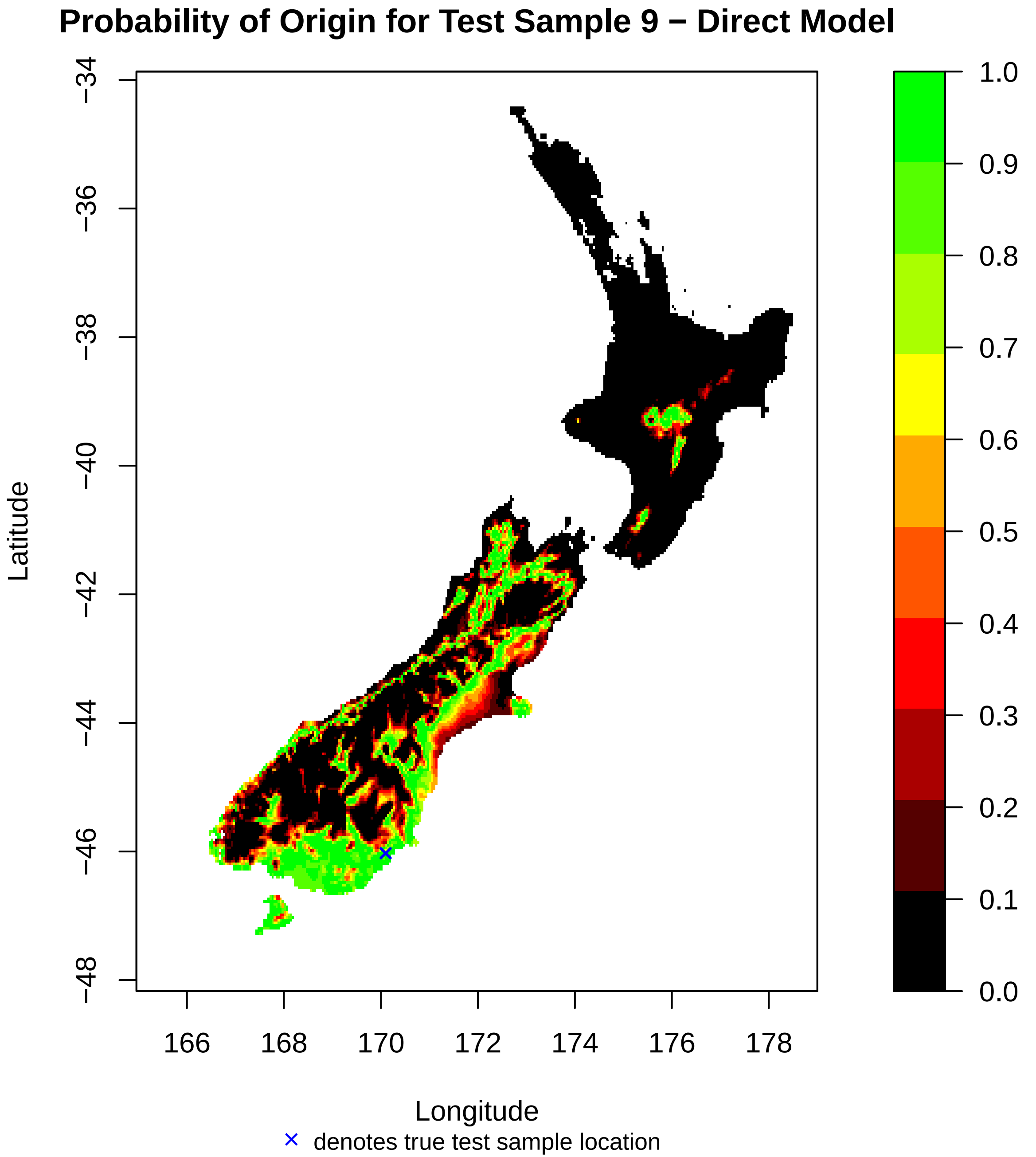

Probability of Origin for Test Sample 9 − Direct Model
Latitude
−34
−36
−38
−40
−42
−44
−46
−48
Longitude
166
168
170
172
174
176
178
1.0
0.9
0.8
0.7
0.6
0.5
0.4
0.3
0.2
0.1
0.0
× denotes true test sample location

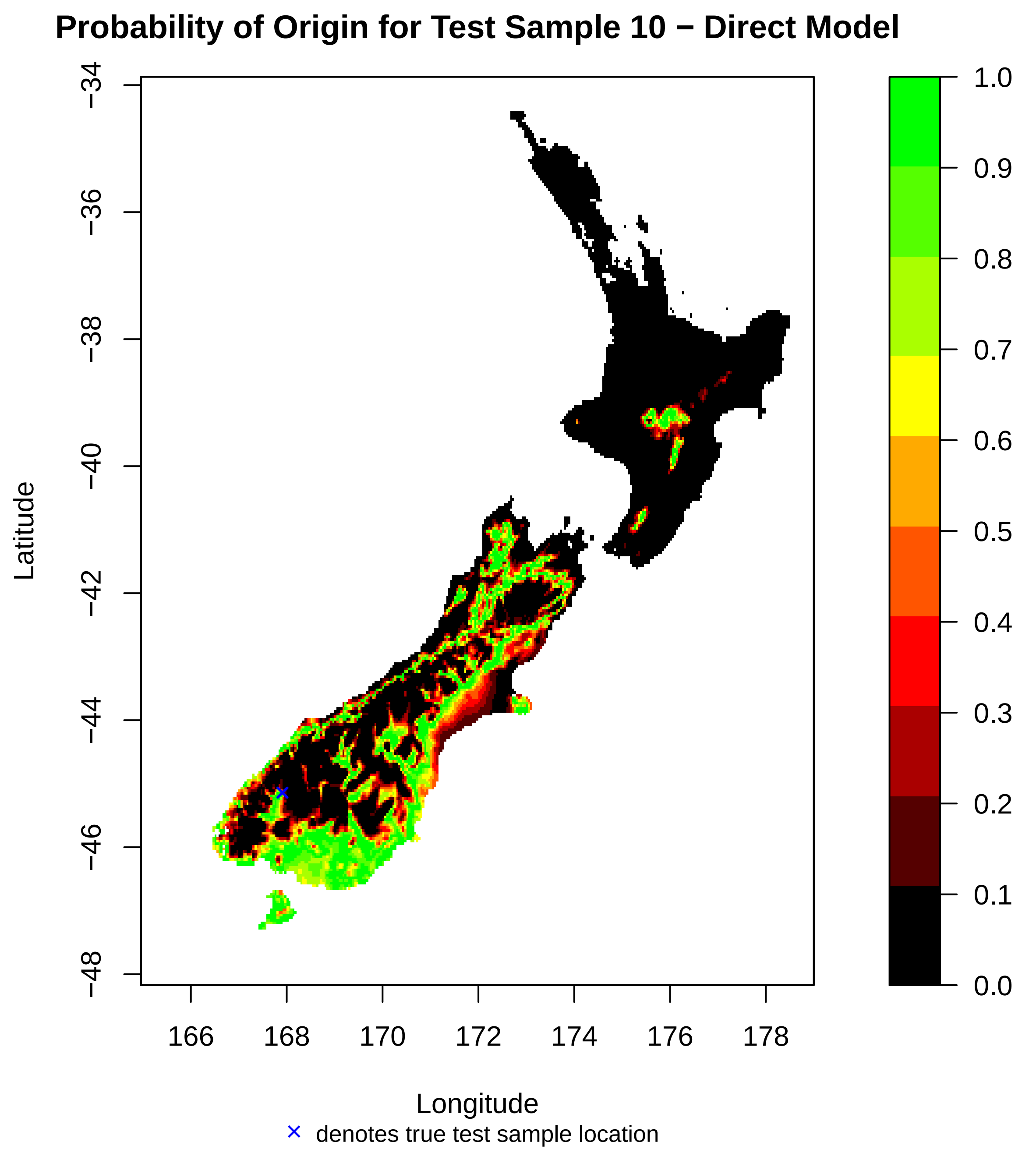

Probability of Origin for Test Sample 10 − Direct Model
Latitude
−34
−36
−38
−40
−42
−44
−46
−48
Longitude
166
168
170
172
174
176
178
1.0
0.9
0.8
0.7
0.6
0.5
0.4
0.3
0.2
0.1
0.0
× denotes true test sample location
