# Supplementary material for: A δ2H Isoscape of blackberry as an example application for determining the geographic origins of plant materials in New Zealand
Source: PLoS One. 2019 Dec 9;14(12):e0226152. doi: 10.1371/journal.pone.0226152 (PMC6901217; doi:10.1371/journal.pone.0226152)
Supplement: S4 File — Red lines indicate the mean value of each comparison. (PDF) [file pone.0226152.s010.pdf]

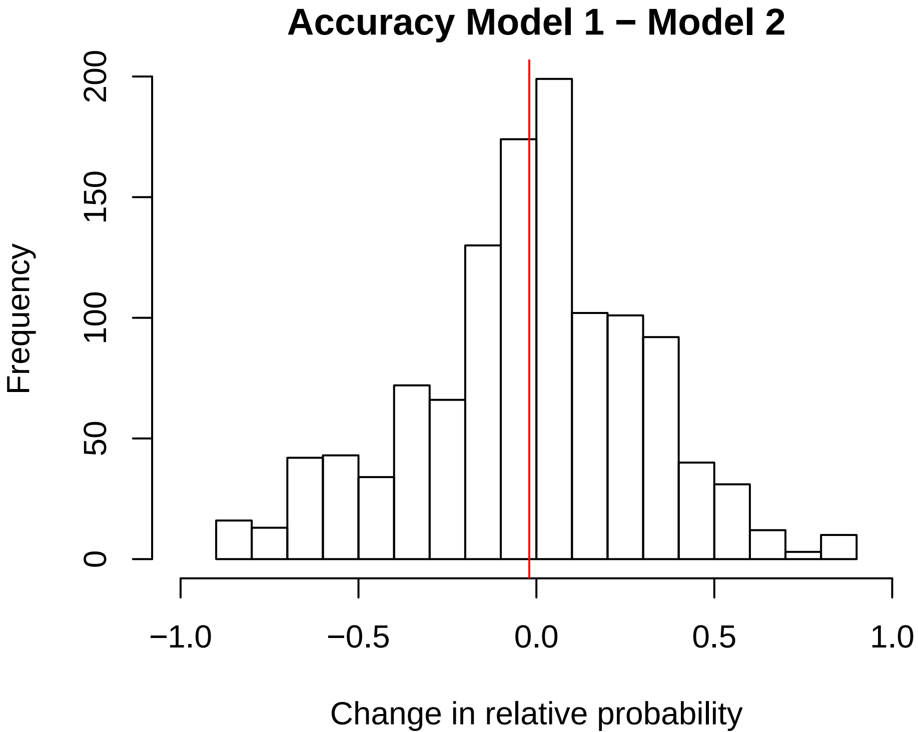

Accuracy Model 1 − Model 2
Frequency
0
50
100
150
200
−1.0
−0.5
0.0
0.5
1.0
Change in relative probability

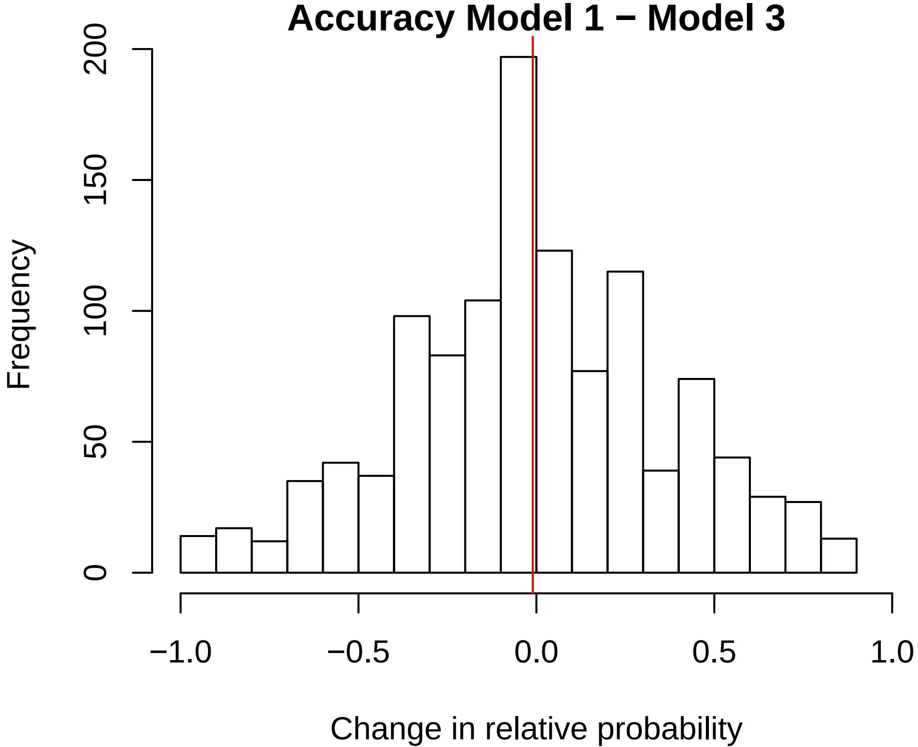

Accuracy Model 1 − Model 3
Frequency
0
50
100
150
200
−1.0
−0.5
0.0
0.5
1.0
Change in relative probability

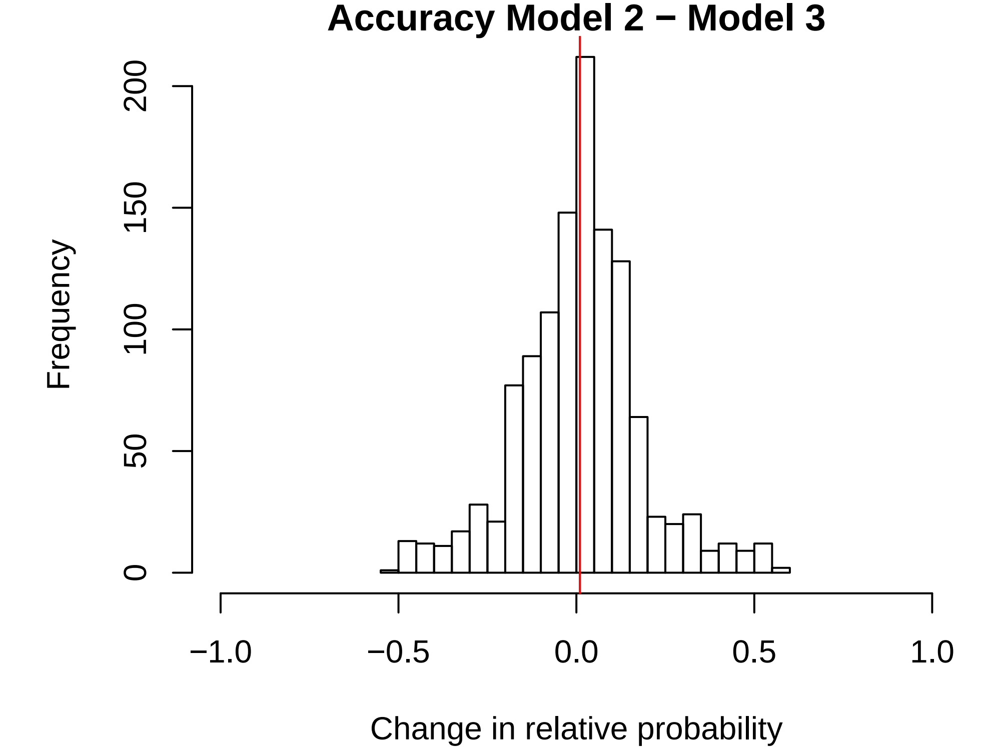
Accuracy Model 2 − Model 3
Frequency
0
50
100
150
200
−1.0
−0.5
0.0
0.5
1.0
Change in relative probability

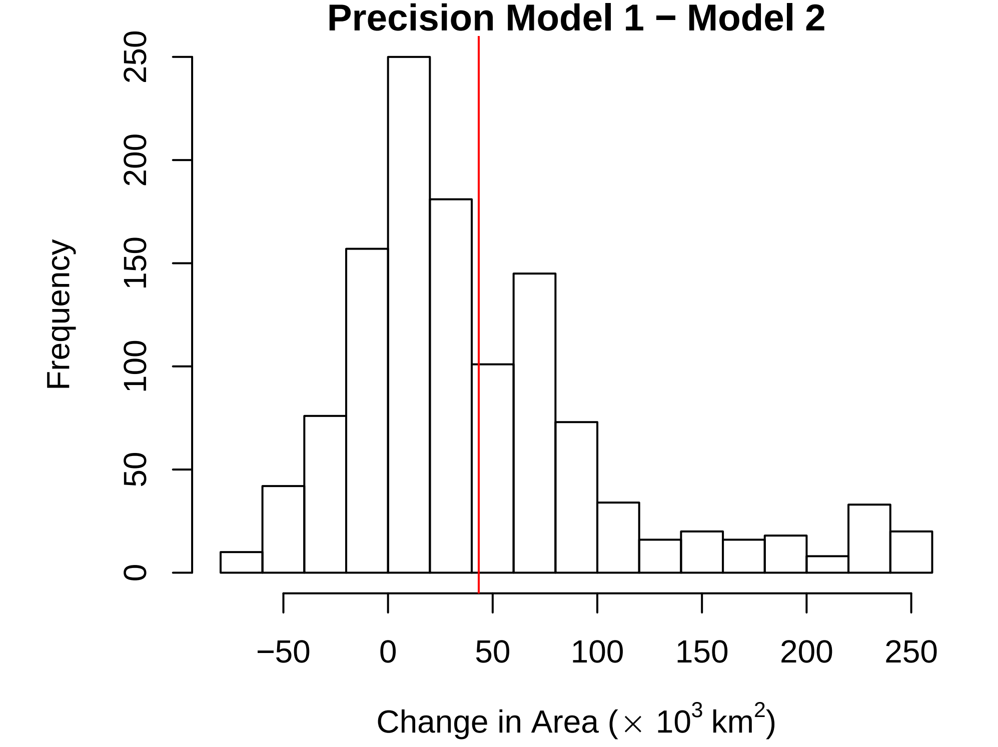

Precision Model 1 − Model 2
Frequency
0
50
100
150
200
250
−50
0
50
100
150
200
250
Change in Area (× 10^3 km^2)

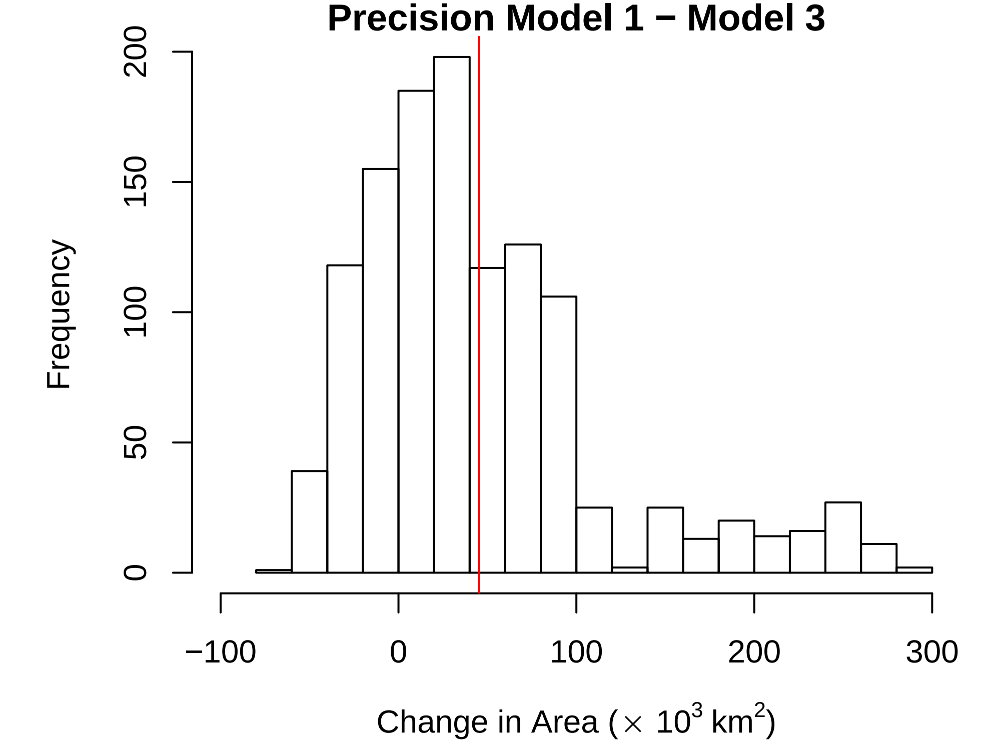

Precision Model 1 − Model 3
Frequency
0
50
100
150
200
−100
0
100
200
300
Change in Area ($\times 10^3$ km^2)

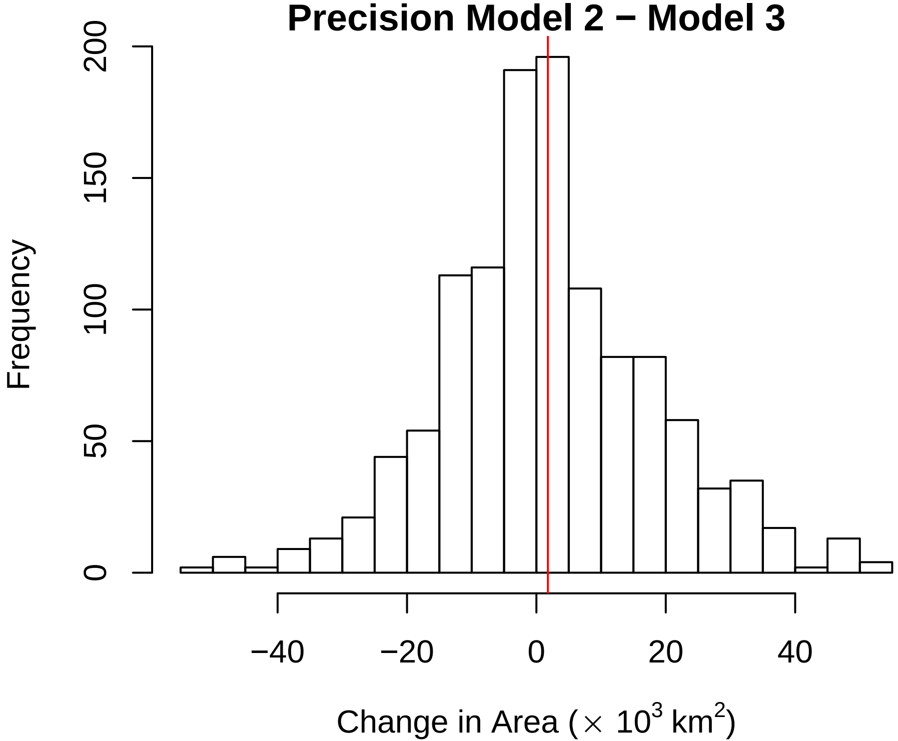

Precision Model 2 − Model 3
Frequency
0
50
100
150
200
−40
−20
0
20
40
Change in Area (× 10^3 km^2)

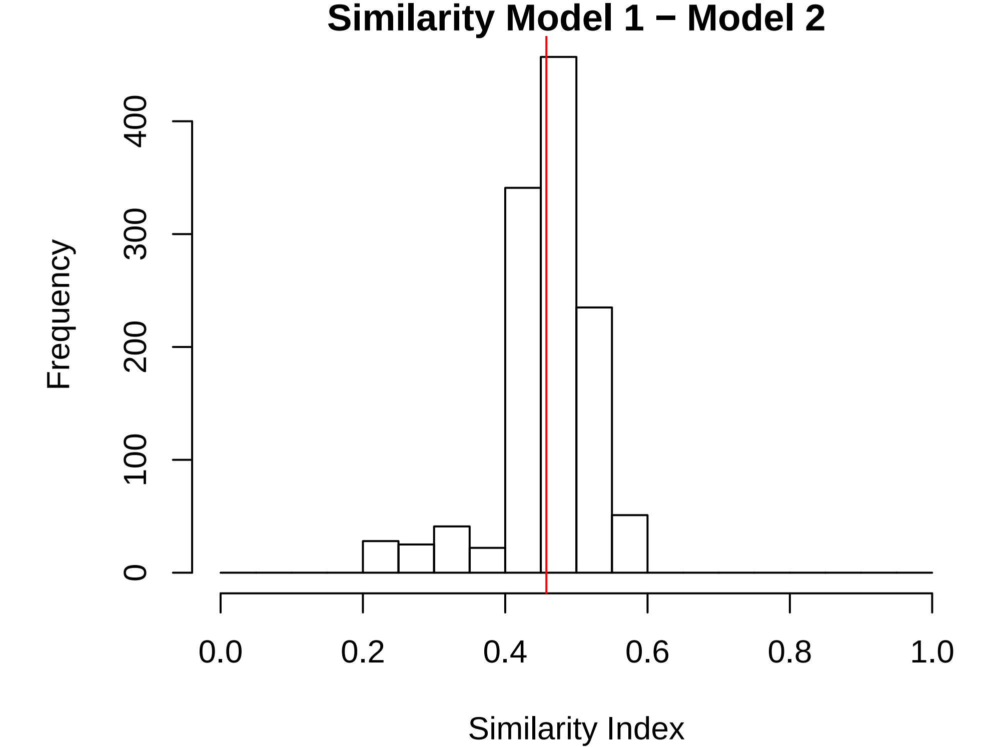
Similarity Model 1 − Model 2
Frequency
0
100
200
300
400
0.0
0.2
0.4
0.6
0.8
1.0
Similarity Index

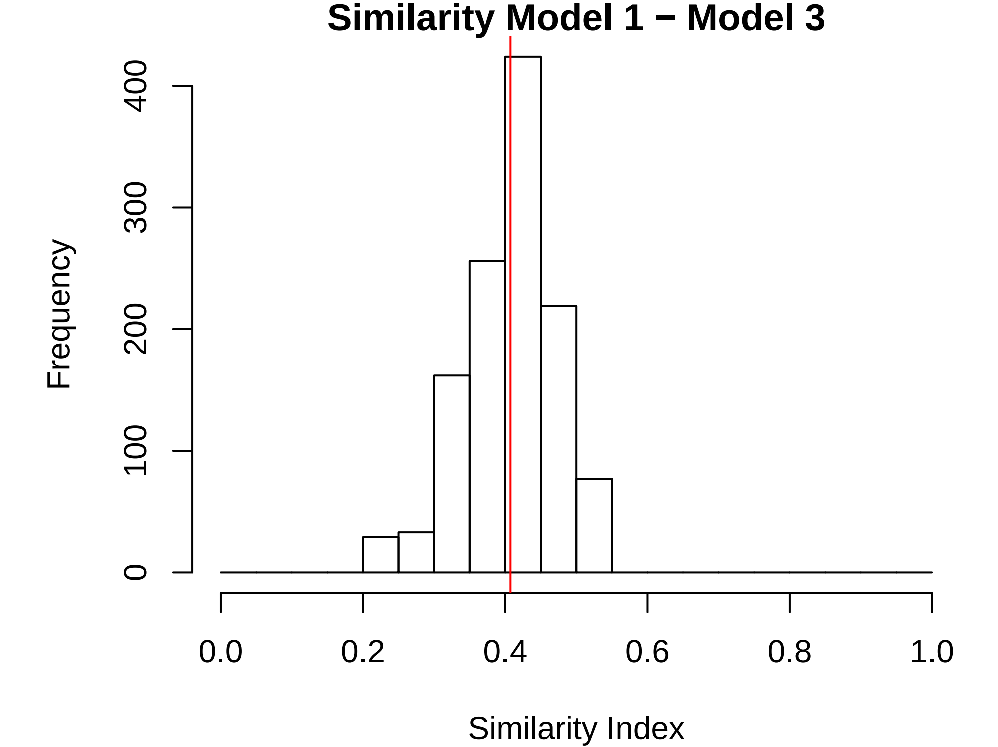
Similarity Model 1 − Model 3
Frequency
0
100
200
300
400
0.0
0.2
0.4
0.6
0.8
1.0
Similarity Index

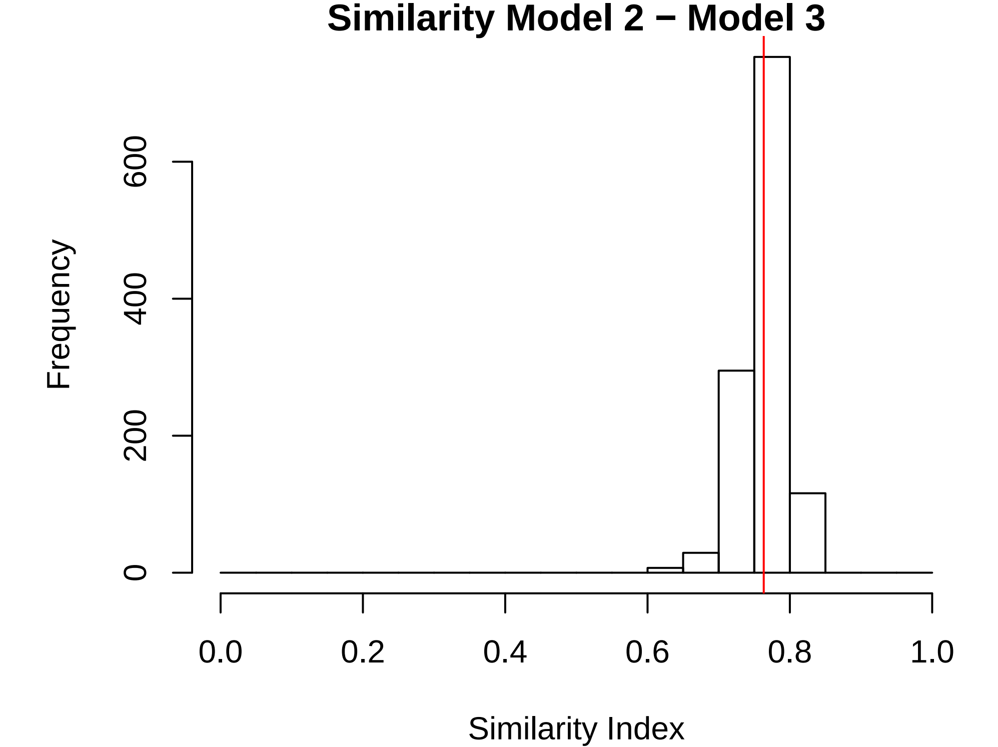
Similarity Model 2 − Model 3
Frequency
0
200
400
600
0.0
0.2
0.4
0.6
0.8
1.0
Similarity Index
